# Exploring the Priorities of Patients with Early Breast Cancer in the United States: A Qualitative Interview Study and Patient-Informed Conceptual Disease Model

**DOI:** 10.3390/cancers17213514

**Published:** 2025-10-31

**Authors:** Ashley Duenas, Zulikhat Segunmaru, Deborah Collyar, Debora Denardi, Claudine Clucas, Klaudia Kornalska, Qixin Li, Chintal H. Shah, Paul Swinburn, Mariana Chavez-MacGregor, Xiaoqing Xu

**Affiliations:** 1 PPD Evidera Patient Centered Research, Thermo Fisher Scientific, London W6 8DN, UK; 2 Oncology Outcomes Research (O2R), Evidence Generation, Publications, and Partnerships, Global Medical Affairs, Oncology Business Unit, AstraZeneca, Gaithersburg, MD 20878, USA; zulikhat.segunmaru@astrazeneca.com (Z.S.); sandy.li1@astrazeneca.com (Q.L.); chintal.shah@astrazeneca.com (C.H.S.); xiaoqing.xu1@astrazeneca.com (X.X.); 3 Patient Advocates in Research, Danville, CA 94506, USA; 4 FORCE—Facing Our Risk of Cancer Empowered, Tampa, FL 33160, USA; 5 Department of Health Services Research, The University of Texas MD Anderson Cancer Center, Houston, TX 77030, USA

**Keywords:** breast neoplasms, patient-centered research, patient engagement, patient experience, qualitative research, quality of life

## Abstract

**Simple Summary:**

Despite recent advances in the treatment of early-stage breast cancer (eBC), the impacts of the disease and its treatment on patients’ lives are poorly understood. This study explored the experiences and unmet needs of women with eBC in the United States. Interviews with 36 women living with eBC revealed how the disease affected them emotionally and psychologically. It also disrupted their social lives and negatively impacted their body satisfaction, daily activities, physical functioning, sexual functioning, and finances. The interviews also revealed communication gaps between healthcare providers and patients. Findings from the interviews and insights from patient experts were used to develop a “conceptual disease model” that summarizes the priorities of eBC patients. This model can help discussions between patients and their physicians on treatment and care. Our findings can also help guide future patient-focused research in eBC.

**Abstract:**

*Background:* Despite recent advances in new therapies for early-stage breast cancer (eBC), the impact of the current treatment landscape on patients’ quality of life remains poorly understood. This study explored the experiences and unmet needs of women with eBC, leading to the development of a patient-informed conceptual disease model (PI-CDM) that summarizes patient priorities. *Methods:* This qualitative study used a step-wise approach: (1) a targeted literature review; (2) draft CDM development; (3) interview guide development; (4) semi-structured interviews with women in the United States with a diagnosis of eBC; (5) thematic content analysis of interview transcripts; (6) patient steering committee insights; and (7) PI-CDM finalization. *Results:* Thirty-six women with eBC (stage I, *n* = 18; stage II, *n* = 11; stage III, *n* = 9) were interviewed between December 2023 and May 2024. Key health concepts included signs and symptoms leading to diagnosis and common treatment side effects. Emotional and psychological impacts were prominent, and 28 participants reported moderate to extremely severe anxiety or depression on the EQ-5D-5L. Other impacts included social life, body satisfaction, daily activities, physical functioning, sexual functioning, and finances. Needs for improved communication from healthcare providers about treatment options and better support were emphasized. These insights, combined with patient steering committee recommendations, resulted in a final PI-CDM. *Conclusions:* This study highlights the substantial burden women with eBC face and provides a framework for future patient-centric research. A CDM developed with patients summarizes the complexity of the eBC experience and can aid discussions between patients and physicians, facilitating shared decision-making to enhance care.

## 1. Introduction

Breast cancer (BC) is the most prevalent cancer in the United States (US) [1,2]. It was estimated that in 2025 there would be 316,950 new cases of invasive BC and 59,080 cases of ductal carcinoma in situ among women in the US [2]. Screening techniques such as mammography and sonography enable most cases of BC to be detected at an early stage [3]. Two-thirds (66%) of women with BC in the US are diagnosed with early-stage breast cancer (eBC) [4,5]. Treating eBC presents unique challenges due to the various factors influencing treatment decisions, including a patient’s age, menopausal status, histological factors, BC subtype, and gene expression profile. The classification of BC into molecular subtypes offers a stratified approach to treatment. In an analysis based on health records from 2011 to 2021 from a US nationwide database, most patients with human epidermal growth factor receptor 2-positive (HER2+) eBC received post-neoadjuvant therapy, while an increasing proportion of patients received neoadjuvant therapy. Almost all patients underwent surgery [6]. Disease management of eBC has improved in recent years because of advances in early diagnosis, the approval of more effective therapies, and the development of better targeted radiotherapeutic techniques, such as partial breast irradiation [7,8,9].

Despite advances in the diagnosis and treatment of eBC, there is limited understanding of its impacts on patients’ day-to-day lives. The current treatment landscape involves prolonged disease management, including aggressive therapies to prevent recurrence, which are often associated with side effects and impacts on short- and long-term quality of life (QoL) [10,11,12,13]. Factors such as age have been linked to lower QoL in women with BC, with patients 42 years old or younger being more susceptible to multiple support needs related to emotional wellbeing, sexual functioning and fertility, work, and survivorship [14]. While this highlights some of the unique challenges of eBC, in-depth qualitative research on the experiences of patients with eBC remains sparse.

Given the growing number of patients diagnosed with eBC and the resulting burden, it is crucial to understand the diverse needs and priorities of this patient population to ensure that treatment, management, and care are patient-focused. Understanding what is important to patients regarding disease-related symptoms, disease impacts, and QoL is essential for monitoring and measuring relevant health concepts that document the patient experience. This study aimed to explore the experiences and unmet needs of women with eBC and develop a patient-informed conceptual disease model (PI-CDM) to organize and visualize the various ways in which eBC and its symptoms affect QoL from the patient perspective.

## 2. Materials and Methods

### 2.1. Study Design

**Figure 1:**
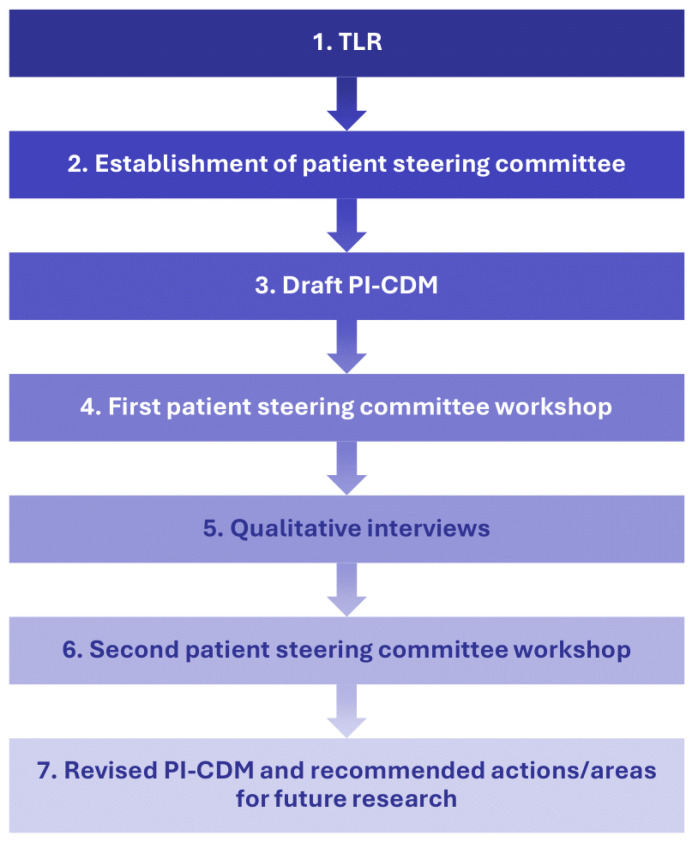
Study flow chart describing a stepwise approach with involvement of a patient steering committee. PI-CDM patient-informed conceptual disease model, TLR targeted literature review.

This qualitative interview study employed a step-wise approach to understand patients’ experience with eBC (Figure 1). Researchers first conducted a targeted literature review (TLR) and drafted a PI-CDM. A patient steering committee, consisting of three patient experts, was established to provide feedback on the TLR results and PI-CDM. The committee’s feedback led to revisions of the draft PI-CDM and informed the development of a semi-structured interview guide that was used in qualitative interviews with patients with eBC. The PI-CDM was further refined based on the results of these interviews.

### 2.2. Targeted Literature Review

A search was conducted in MEDLINE/PubMed and Embase for research articles published between 2016 and 2022 to identify patient-related eBC concepts, with a focus on signs and symptoms, treatment side effects, and impacts on daily life. Most articles identified in the literature search were quantitative observational studies. Therefore, information from patient websites, guidelines, and clinical trial databases was also reviewed [15,16]. The TLR informed the creation of a list of concepts that were organized into a preliminary CDM. These concepts included proximal and distal impacts—those more directly or indirectly related to eBC and effects of its treatment [17]. The impacts encompassed physical functioning and mobility, daily activities and lifestyle, emotional and psychological wellbeing, social and sexual functioning, work and school impacts, and financial stress and difficulties.

### 2.3. Patient Steering Committee

Engaging patients during and after the drug development process is crucial for ensuring therapies support patient-relevant outcomes [18]. In the real-world setting, patient engagement can support research that aligns with patient priorities, such as relevant needs and concerns [18,19,20]. In the present study, we established a patient steering committee consisting of three patient experts known to the study team, two of whom represented fellow BC patients in the FORCE patient advisory group [21]. The patient experts on the committee participated in two workshops. In the first workshop in April 2023, the committee helped contextualize the TLR findings and provided insights that were incorporated into the qualitative interview guide. In the second workshop, the committee provided input on the findings from the qualitative interviews, including their accuracy and any unaddressed elements. The committee also helped ensure the PI-CDM’s relevance to eBC and offered suggestions on its potential applications. Following the second workshop, the PI-CDM was refined and published [16].

### 2.4. Qualitative Interviews

Qualitative one-on-one interviews were conducted at a single point in time via audioconference to understand participants’ experiences with eBC, including their treatment journey, symptom burden, impacts on QoL, and unmet needs.

#### 2.4.1. Ethics

The study protocol, informed consent form, and interview guide were submitted to the Advarra Institutional Review Board, which determined that no ethical approval was required.

#### 2.4.2. Participant Recruitment

A third-party recruitment vendor, Global Perspectives, recruited a convenience sample of participants in the US using quotas to ensure balanced inclusion across BC subtypes, disease stages, and sociodemographic backgrounds. Global Perspectives identified potential participants through their patient panel database, social media, patient associations, and physician referrals, using standardized recruitment messaging to advertise the study. All recruitment procedures complied with the Health Insurance Portability and Accountability Act.

#### 2.4.3. Eligibility Criteria

Individuals interested in participating in an interview contacted Global Perspectives to learn more about the study. To be eligible, participants had to be English-speaking adults (age ≥ 18 years) living in the US with a self-reported diagnosis of eBC (stage I, II, or III) within the past 12 months, specifically one of three subtypes: hormone receptor-positive (HR+)/HER2-negative (HER2-), HER2+, or triple-negative breast cancer (TNBC). Participants with cognitive impairments, hearing difficulties, visual impairments, or severe psychopathology were not eligible and were thus excluded.

#### 2.4.4. Pre-Interview Procedures

Eligible participants received an email to complete an online pre-interview survey with sociodemographic and medical background questions (Supplementary Methods). The first page of the survey directed participants to a consent page with standardized text explaining the study and what was involved. After participants provided consent, they completed the pre-interview survey, which included questions on sociodemographic and clinical characteristics such as age, race and ethnicity, date of diagnosis, BC stage and subtype, treatments received, and comorbid health conditions. The pre-interview survey also included the EQ-5D-5L [22], a patient-reported outcome (PRO) instrument that assesses health-related QoL. The EQ-5D-5L consists of five domains (mobility, self-care, usual activities, pain/discomfort, and anxiety/depression), as well as a visual analog scale (VAS) for participants to rate their health from 0 (the worst health you can imagine) to 100 (the best health you can imagine) [22].

#### 2.4.5. Interview Procedures

Interviews lasted up to 60 min and were conducted by trained female qualitative researchers with a Master of Science or Public Health degree (AD, BH, DT, DSE) as employees of PPD Evidera Patient Centered Research, part of Thermo Fisher Scientific.

The study team developed an interview guide based on findings from the TLR and patient steering committee feedback. It included open-ended questions to explore participants’ experiences of diagnosis and treatment, symptoms, treatment side effects, impacts, and unmet needs relating to eBC. The interview guide was tested during the first three interviews by a senior team member (AD), who conducted these interviews. A team debriefing was completed after these first interviews. The wording of the questions was amended to increase clarity and the number of questions reduced to allow more in-depth answers (see Supplementary Methods).

Interviews were audio recorded and transcribed by Global Perspectives. Each audio file was transcribed by one transcriber and independently quality controlled by another transcriber to ensure a good quality of transcripts. An additional quality check to remove any personally identifiable information was performed by the project team. Only the interviewer and participant took part in the interview. The participants did not know the interviewers before the interviews. At the start of each interview, the interviewer introduced themself as an employee of Evidera (a consulting company specializing in health research) and outlined the study goals. The interviewers took notes during the interviews. Senior members of the study team (AD and CC) performed quality checks on transcripts. These quality checks involved reading the transcripts to assess that the study procedures were being followed according to the study protocol and to ensure that the interviewers were following good interviewing practice (such as using follow-up probes to guide the interview and avoiding leading questions). Following the quality checks, the senior members of the study team provided feedback to the interviewers.

No repeat interviews were conducted. Participants did not comment on the transcripts or provide feedback on the study findings. Participants received a $165 gift card for completing the pre-interview survey and interview.

### 2.5. Data Analysis

#### 2.5.1. Pre-Interview Survey Sample Characteristics

Quantitative responses to the pre-interview survey were analyzed with SAS v9.4 (SAS Institute, Cary, NC, USA). Descriptive statistics were calculated (categorical variables: count and percentages; continuous variables: mean and standard deviation [SD] or median, interquartile range [IQR], min, and max). No statistical tests were performed; the data were only used to describe the sample of participants included in the study.

#### 2.5.2. Qualitative Data

Interview transcripts were analyzed using a thematic content analysis method [23,24]. ATLAS.ti 22 was used to organize the data [25]. A coding framework (Supplementary Methods) was developed using the interview guide and adjusted according to themes and concepts identified directly from the patient interview data. The coders applied the constant comparative method to identify new codes and refine existing ones by iteratively comparing new data with previously coded data.

Three coders (EW, DT, and DSE; all female employees of Evidera, part of Thermo Fisher Scientific) were trained on the coding framework and independently coded the first two interview transcripts. All codes were then compared and carefully reviewed by senior researchers (AD and CC) and, where differences occurred, reconciled during a reconciliation meeting. Subsequent transcripts were coded by the same three coders. Any further disagreements in the coding process were resolved by senior researchers (AD and CC). All coding was subject to rigorous quality control by the same senior researchers, who read the transcripts and assessed the application of codes. The quality control step was implemented to ensure that the codes had been applied consistently and accurately, i.e., the code labels corresponded to the correct passage of text and all relevant codes were applied.

No participant check of the coded data was conducted; however, the study results were reviewed by the patient steering committee.

#### 2.5.3. Saturation of Concepts

In qualitative interviews, “saturation” is the point at which no substantially new information continues to emerge beyond what has already been obtained [26,27]. The Food and Drug Administration requires evidence of saturation in qualitative research [28]. In this study, interview transcripts were sequentially divided into groups of six. As each new set of interview transcripts became available, the number of new concepts was analyzed to determine whether saturation had been achieved.

## 3. Results

### 3.1. Participant Characteristics

Forty-two women with eBC from the US were contacted to take part in this study. Two of these women were excluded based on their diagnosis dates, and three others were excluded without being screened because the target number of participants had already been interviewed. Another participant withdrew from the study partway through the interview. The remaining 36 women completed interviews between December 2023 and May 2024.

The median age of participants was 50.5 years (IQR: 42.75–56.0). Twenty-two participants were White, eight were Black or African American, two were Native Hawaiian or other, and one was Asian (Table 1). Six participants identified as Hispanic or Latino. The participants’ education ranged from secondary school to postgraduate education, with 27 participants having at least some college education. In terms of clinical characteristics, 18 participants had stage I disease, 11 had stage II, and 7 had stage III. Fourteen participants had HER2+ tumors, 12 had TNBC, and 10 had HR+/HER2- tumors. Five participants reported *BRCA* gene mutations. At the time of the interview, the mean (SD) time from diagnosis was 10.1 (10.24) months.

Thirty-five participants completed the EQ-5D-5L. Most participants experienced anxiety and depression, with 28 participants reporting being moderately, severely, or extremely anxious or depressed. Pain and discomfort were also experienced by more than a quarter of participants, with 10 having moderate or severe pain and discomfort. The majority of participants (*n* = 25) reported no mobility problems, and no participants reported any severe or extreme problems with mobility or self-care. Due to the small sizes of the subgroups, there was no evidence of differences by disease stage or subtype. The mean VAS score across all participants was 67. Mean VAS scores were lowest (worst) in participants with stage II disease (63) and HR+/HER2- tumors (63).

### 3.2. Qualitative Interview Findings

Results of the qualitative interviews were organized into the following themes: (1) experiences with eBC diagnosis and genetic testing, (2) signs and symptoms of eBC, (3) treatment experiences for eBC, (4) impacts of eBC, and (5) unmet needs associated with eBC. Illustrative participant quotes are available in Tables S1–S3 in the Supplementary Materials. This qualitative study is reported in line with the COnsolidated criteria for REporting Qualitative research (COREQ) [29] (Table S4).

#### 3.2.1. Experiences with eBC Diagnosis and Genetic Testing

Study participants were asked to describe their diagnostic journey with eBC. Most participants described mammography, biopsy, and/or ultrasound procedures that led to an eBC diagnosis. Some participants also described having an MRI/CT scan or bloodwork. Sixteen participants noticed a lump via self-examination or during a routine medical examination.

More than half of the participants reported having taken a genetic test for BC. However, a few of these participants did not know the specific type of genetic testing they had received. Additionally, three participants mentioned taking so many diagnostic tests that they were unsure if they had received a genetic test. Despite this, most participants who completed a genetic test could articulate its purpose and understood that it related to heredity risk of BC and other cancers.

#### 3.2.2. Signs and Symptoms of eBC

The qualitative interviews identified 14 eBC signs and symptoms (Figure S1 in the Supplementary Materials). Signs and symptoms endorsed by more than one participant were lump on breast or underarm, breast or nipple pain, energy-related symptoms, nipple or breast inflammation or swelling, nipple discharge, skin changes, nipple retraction, and weight loss. Saturation of concepts is presented in Table S5. Eight of the 14 signs and symptoms were elicited in the first six interviews. All other signs and symptoms were identified in the first four groups of interviews (i.e., in the first 24 interviews), with the exception of a single report of dimple in the breast in the final interview.

Signs and symptoms varied considerably between participants and across disease stages. Commonly reported signs and symptoms included a lump, breast or nipple pain, and energy-related symptoms, with some participants experiencing all three symptoms simultaneously.

“*Okay. So, the early onset breast cancer, I had a strange itching in my breast and that was the pressure of the lump. I was exhausted all the time. I didn’t know why. I had pain; it felt like my underarm was on fire all the time, like shooting pain.*”

(P12_Stage III_HER2+)

Nine participants—four with HER2+ eBC and five with TNBC—did not endorse any signs or symptoms. Seven of the participants reporting no signs or symptoms had stage I disease. Participants with stage I disease were less likely than those with stage II or III disease to report breast or nipple pain and energy-related symptoms, such as fatigue, tiredness, and lethargy (Figure S2). Endorsement of signs and symptoms did not differ markedly by cancer subtype.

#### 3.2.3. Treatment Experiences for eBC

Participants described surgery (such as lumpectomy/breast-conserving surgery, single mastectomy, or double mastectomy) as a common treatment. Twenty-Four participants confirmed undergoing surgery for eBC before the interview, and 15 participants had a surgery planned at the time of the interview (Figure S3).

Side effects from cancer treatments included pain and discomfort (*n* = 28), energy-related impacts such as fatigue or tiredness (*n* = 25), weight or appetite loss (*n* = 14), cognitive impacts (*n* = 13), hair loss (*n* = 11), and gastrointestinal issues (*n* = 10) (Figure S4).

Pain and discomfort were attributed to surgery, radiation therapy, or chemotherapy. Nearly half of those who experienced pain and discomfort from treatment described it as intense or severe, with one woman unable to get out of bed because of treatment-related pain in her bones and joints.

“*The first medication that I was on, it was a lotta days I couldn’t really get out the bed because it was causing my bones and joints to hurt extremely bad. So, she switched it and I’m on a new one, and now I just take it ‘cause I see that, no matter what, my body is still gonna ache. I still don’t sleep. So, I feel like it’s just something that I have to accept now.*”

(P8_Stage I_HR+/HER2-)

Energy-related side effects including fatigue, lack of strength, and low energy were severe for most participants who experienced them. One participant expressed, “*I think a lot of that was a level of exhaustion that it was like I couldn’t think straight*” (P28_Stage I_TNBC). Another participant described napping to cope with tiredness, while a third lacked energy to do the dishes. In a few cases, energy-related side effects were specifically attributed to radiation therapy, hormone therapy, chemotherapy, immunotherapy, or surgery.

Participants experienced appetite loss and/or weight loss, with some reporting weight loss of 20 pounds or more or having to force themselves to eat. Some participants attributed their appetite loss or weight loss to chemotherapy; one participant specifically attributed her appetite loss to being unable to taste anything because of chemotherapy. Two participants specifically attributed their appetite loss to radiation therapy.

“*When I noticed during the radiation you really don’t have that much of an appetite. I mean, there was a little bit of weight loss there, and it was frustrating ‘cause you wanna take care of everything but, at some point, it was depressing and didn’t really wanna do anything except for watch TV or something.*”

(P14_Stage I_HER2+)

Four participants reported medication-related weight gain; two of these participants attributed their weight gain to hormone therapy. One of them called it “…*a side effect of the medicine.* […] *The hormone one. It’s one of the main side effects*” (P2_Stage II_HR+/HER2-). The other participants attributed weight gain to “IV therapy” or did not specify the type of medication they were taking.

Thirteen participants reported cognitive impacts, including memory problems, brain fog, and issues with focus and concentration. Some participants attributed their cognitive difficulties to chemotherapy, with two using the phrase “chemo brain” and one saying, “*The first year, I couldn’t really remember a lot*” (P8_Stage I_HR+/HER2-). Another participant described how their “…*ability* […] *to multi-task, to focus, to attend to information topics, remember them, my ability to process things step-by-step, follow through on things,* […] *stay organized, all that is more difficult*” (P28_Stage II_TNBC).

Nearly half of the participants who experienced hair loss because of their eBC treatment specifically attributed it to chemotherapy. Two participants said their hair came out “in clumps.” One participant described starting to lose her hair, “*…[it] bothers me a lot. It’s just terrible. Especially for the women, I know it’s one of most… one of the worst things that is very hard and not easy to accept it, but what can I do? It is very painful, but I have to take it*” (P13_Stage I_HER2+).

Gastrointestinal side effects were experienced by 10 participants and included nausea vomiting, diarrhea, constipation, and hemorrhoids. In a few instances, gastrointestinal side effects were attributed to chemotherapy or surgery. Three participants described the gastrointestinal side effects as severe, with individual participants mentioning “*projectile vomiting [that was] violent and rough and hard*,” having “*a bad case of nausea*” and not being able to eat without throwing up, and having “a lot” of constipation.

No clear pattern of differences in endorsement of side effects was observed according to disease stage or cancer subtype (Figure S5).

#### 3.2.4. Impacts of eBC

The interviews identified 14 impacts (Figure S6). Saturation of impacts is documented in Table S5; 13 impacts were reported in the first six interviews. One new impact emerged in the second set of six interviews and none in subsequent interviews.

The most commonly reported areas of impact included emotional and psychological, social life, body satisfaction, daily activities, and physical functioning. Participants with stage III disease frequently reported disruption of daily activities, including work and school (Figure S7). Impacts on intimacy were commonly discussed by participants with stage II disease. Participants with TNBC had a greater tendency to report impacts on cognitive functioning, compared with participants with HR+/HER2- or HER2+ eBC. Otherwise, endorsement of impacts was consistent across cancer subtypes.

**Figure 2:**
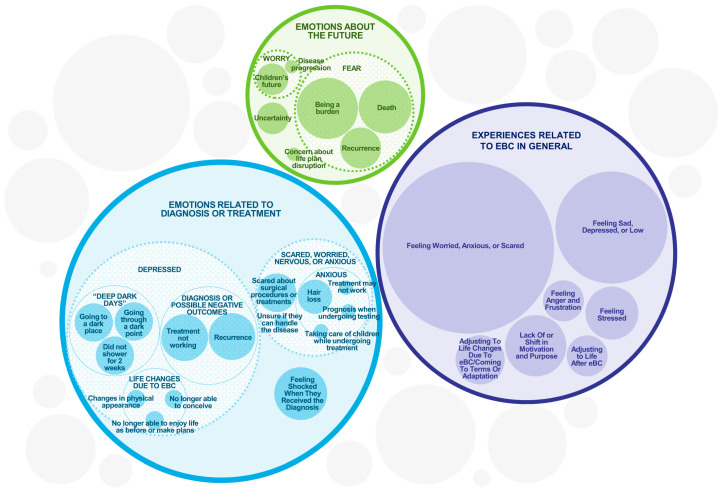
Emotional and Psychological Experiences of eBC Patients. The sizes of the circles are illustrative of the frequency of each concept while accommodating their sub-themes, but are not intended to be an exact numerical representation.

The emotional impact of eBC was substantial across the entire sample (Figure 2). All 36 participants experienced some form of emotional or psychological impact, mostly manifesting as feelings of worry, anxiety, or fear (*n* = 32) or sadness, depression, or low mood (*n* = 21). Eight participants expressed fear of recurrence. One participant described a significant and long-term emotional impact.

“*…I felt like I was less than a woman, you know, losing my hair and losing my breast, getting everything taken out. All that is what makes you a woman, and I no longer had that. It made me… I don’t know. I was really depressed and I had anxiety. It still makes me emotional thinking about it. It just altered my life.*”

(P8_Stage I_HR+/HER2-)

Other participants reported a lack of or shift in motivation and purpose, feeling shocked when they received their diagnosis, stressed, or angry and frustrated. The participants listed several reasons for the emotional and psychological impacts, such as the initial diagnosis and what they experienced or not understanding why it was happening to them.

The experience of being diagnosed with eBC was overwhelming for some, with one participant calling it a “whirlwind” and describing how “*all of a sudden, [I] went from thinking my life was fine, no issues, not any problems whatsoever, and then all of a sudden I was diagnosed with breast cancer. We have scheduled my double mastectomy for this coming Monday, and I’m really scared*” (P20_Stage II_HR+/HER2-). Another participant described it as an “emotional roller coaster” (P14_Stage I_HER2+).

Across 26 participants, social interactions were impaired in several ways. This included participants socializing less as a result of them focusing on taking care of themselves. Participants described limiting the types of social engagements they were willing to participate in, declining social invitations, and not wanting to be a burden to family. Three participants explicitly described avoiding having conversations dominated by the topic of cancer. One participant explained that “…*everything in my life has kinda come to a stop right now*” (P15_Stage I_HER2+). While two participants reported negative impacts on personal and intimate relationships, two others noted that eBC led to new friendships with individuals who shared similar experiences.

Surgical and non-surgical treatments had a profound effect on how participants viewed their bodies, with many reporting impacts on body satisfaction (*n* = 25), including an altered self-perception of their physical appearance. One participant expressed, “*Well, it’s a little scary because us women are built a certain way and if our image is altered, then we don’t feel as good about ourselves as we did*” (P4_Stage III_HER2+). Participants reported impacts on body satisfaction regarding surgical scars or surgical area, hair loss, changes in the appearance of their breasts, and weight loss.

Participants’ experiences with eBC added difficulty to their daily lives. Participants (*n* = 19) described impacts on daily activities, including housework, grocery shopping, washing and dressing, and driving. Reasons for difficulties with daily activities included pre- or post-treatment tiredness; treatment-related loss of energy, pain, or arm weakness; the surgical incision limiting strenuous activities; and other physical limitations following surgery. Impacts on physical functioning (collectively reported by 17 participants) included strenuous activity, lifting, and movement being restricted due to surgery; decreased activity levels due to tiredness and reduced strength; general weakness; and decreased mobility due to side effects.

Thirteen participants reported impacts on sexual functioning, including reduced desire and lack of interest in sexual activities due to factors such as preoccupation, sadness, and not feeling attractive. Some participants were having less sex with their partner or not having sex at all. When discussing the impact on their sexual functioning, one participant said, “…*I’m not interested in any of that. I think I’m just too sad to be able to do anything like that*” (P4_Stage III_HER2+). Another participant expressed how she “…*[didn’t] really have too much of the desire*” (P34_Stage I_TNBC). And a third participant stated that she was “*not feeling really sexy right now*” (P15_Stage I_HER2+).

Of the participants reporting lifestyle impacts (*n* = 12), half reported an inability to maintain their exercise routine or their vigor, and the same proportion reported an impact on their ability to live life as they used to. Other lifestyle impacts included food intake such as eating more because of stress or eating less while on treatment, “shutting down” and avoiding activities and interactions with others, and being unable to engage in tasks that involved repetitive motions.

#### 3.2.5. Unmet Needs Associated with eBC

When discussing their experiences of diagnosis and treatment, participants described unmet needs relating to eBC support. This included the need for support with logistical issues (such as insurance delays and appointment scheduling), as well as the need for an emotional support system throughout the treatment journey. One participant conveyed a need for improved communication with her healthcare provider about treatment (explaining why treatment was required, discussing expectations around side effects).

“*The radiologist, they didn’t tell me about the burning that might occur… a lack of letting me know why and what for, and they didn’t explain anything. It was just like, do this or do that, and I didn’t… I had asked all the questions; no one would take the time to say, ‘This is why we’re doing this’.*”

(P14_Stage I_HER2+)

One participant described her experience of eBC as “super lonely” (P12_Stage III_HER2+) because her family could not be with her during chemotherapy due to COVID. Another participant reported having insufficient resources, such as wigs and other things that could improve self-confidence. Other unmet needs related to financial and insurance planning. For some participants, inadequate insurance coverage forced them to make difficult financial decisions.

#### 3.2.6. Final PI-CDM for eBC

**Figure 3:**
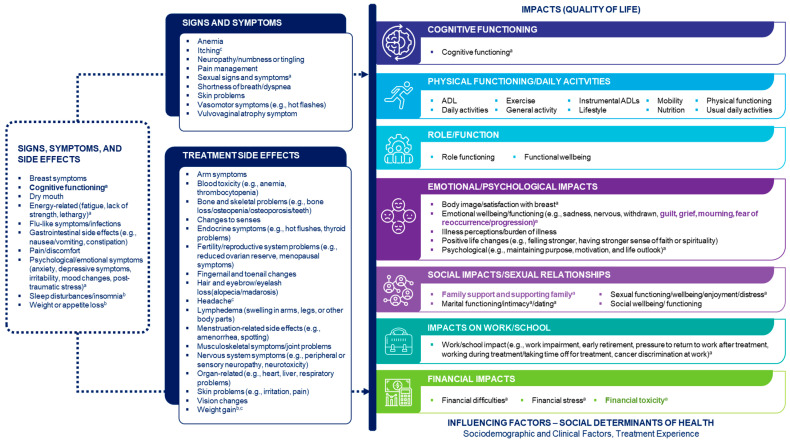
Finalized Patient-Informed Conceptual Disease Model of Early-Stage Breast Cancer. The model does not specifically capture biological aspects of early-stage breast cancer. Concepts in the ‘Signs, symptoms and side effects’ box were specified as both signs/symptoms and side effects. Concepts shown in bold were added after discussion with the patient experts. ^a^ Emphasized as particularly important by the patient experts. ^b^ Also mentioned by the patient experts as an impact. ^c^ Derived from the qualitative interviews. ADL activities of daily living, QoL quality of life.

The PI-CDM for eBC was finalized based on findings from the qualitative interviews and insights obtained during a second patient steering committee workshop on June 6, 2024. The qualitative interviews confirmed concepts identified from the TLR and discussions with patient experts and identified additional side effects and symptoms. During the second workshop, the patient steering committee highlighted topics that warrant further research, including the unmet needs of patients with eBC (such as problems with insurance coverage for mastectomy and breast reconstruction) and the need for support with family planning. The committee further highlighted how patients’ concern about their own family is an important issue that often remains unaddressed in research. The final PI-CDM is shown in Figure 3.

## 4. Discussion

Through engagement with patient experts and in-depth qualitative interviews, we have developed a comprehensive PI-CDM that summarizes what is important to people with eBC. Engaging patients is crucial for ensuring that the patient voice is heard and integrated into research. Our approach aligns with best practices for patient engagement [30] and the National Health Council’s blueprint for developing patient-centered core impact sets, which are patient-selected lists of the most important impacts of diseases (or their treatment) [31,32]. Our results provide empirical qualitative evidence of patient experiences with eBC, which can provide utility for patients, researchers, and healthcare providers [33].

The results from this qualitative study indicate how patient experiences of eBC vary considerably. Within these experiences, however, there is a common thread of emotional and psychological impacts that were experienced by all participants, including fear of recurrence. Many participants also reported impacts on their body image, social lives, and ability to perform daily activities. Previous research has shown that eBC symptoms and impacts can have further downstream effects on patients’ daily lives. For example, fear of recurrence is associated with greater healthcare utilization and worsened emotional distress [34]. Cancer-related body image issues [35] and social isolation [36,37] are linked to impaired job performance and other employment issues, which can lead to financial difficulties. Moreover, challenges in performing daily activities can result in employment difficulties, loss of independence, and changes in lifestyle [38,39].

Major themes that emerged from the analysis of participants’ unmet needs included a lack of emotional support and issues with the delivery of care. This mirrors an earlier cross-sectional study of 944 patients with different cancers, in which the subsample of patients with BC frequently reported unmet psychological needs and logistical needs such as for help getting information and navigating the healthcare system [40]. Other research indicates that unmet needs are associated with poor mental health and low QoL in cancer survivors [41,42,43], emphasizing the urgent need to address these issues.

This PI-CDM is the first comprehensive CDM for eBC. It was informed by the Wilson and Cleary conceptual model of patient outcomes [17] and was developed and finalized based on information from empirical research, insights from patient experts, and qualitative interviews with patients with eBC. While the PI-CDM shows common themes experienced by patients with eBC, it also highlights the heterogeneity of the patient experience beyond symptom burden. Understanding psychosocial determinants or drivers of health in patients with eBC may help to explain health inequities. A recent scoping review concluded that there was strong evidence linking social determinants of health with health inequities in oncology and found that the most frequently reported link between social determinants of health and health equity was the one between social exclusion and discrimination and access to treatment [44]. This has implications for patients with eBC, given that over two-thirds of our sample reported social impacts.

The PI-CDM offers a framework and a common language to support discussions between patients and their physicians, thus establishing patient expectations of the eBC experience at diagnosis (potentially mitigating anxiety) and facilitating shared decision-making about treatment. This model is also intended to serve as the starting point for future research in identifying relevant patient-informed concepts for exploring the use of PRO measures. The evaluation of clinical trial participants with routine PRO measures could identify specific needs, particularly emotional ones, and trigger potential referral to social services or counseling or other services, as needed. Further work is encouraged to identify and assess the adequacy of existing PRO measures, or when developing new PRO measures, based on the list of concepts. The findings based on real-world experiences of women with eBC are also informative when considering what is important for patients in the context of developing new treatments for eBC. Finally, a better understanding of eBC experiences can help identify gaps and shortcomings in existing support systems and thereby guide improvements aimed at optimizing patient outcomes.

While the PI-CDM provides a framework of patient priorities, further work is recommended to validate and apply these results within different contexts of use. For example, patient experts within the study emphasized the importance of the PI-CDM in the healthcare setting, where it might potentially inform clinical guidelines and educational programs for healthcare professionals. Such an approach would require multi-stakeholder consultation to refine the PI-CDM so that it can be applied appropriately. Reviewing the PI-CDM with a larger sample could be one step towards refining the breadth of concepts identified from this qualitative study.

To our knowledge, this is the first qualitative study that explores the patient journey from the perspective of individuals with eBC. This study used qualitative methodologies to identify themes and new hypotheses that may be transferable to similar populations and contexts. The results should be interpreted within the diverse sample of women interviewed and may not apply to populations not represented in this sample, such as men with BC, individuals with advanced or metastatic BC, or races and ethnicities not included in this study. Our study results are based on US participants and may not be transferable to populations in other countries with significantly different healthcare systems. Future research should focus on the experiences of these unrepresented populations. Notwithstanding these limitations, we made efforts to capture representation of different disease stages and molecular subtypes, and our sample size was sufficient for concept saturation to be achieved.

The treatments patients receive for eBC and their diverse impacts appear to be critical determinants of the patient experience. Although this study provides important insights into the treatment journey amongst eBC patients, it was beyond the scope of this research to explore differences in experience by treatment type. We did however examine experiences by disease stage and molecular subtype, which should be explored further in larger studies. Future research should also investigate how experiences differ according to treatment modality.

## 5. Conclusions

This study demonstrated that women diagnosed with eBC experience a significant burden and major impacts on their daily lives. The qualitative interview results provide insights into patients’ complex and varied experiences of eBC treatment, impacts, and overall disease management. By identifying unmet needs and treatment concerns, this study highlights the continuing need for more research into the varied experiences of patients with eBC throughout their treatment journey. To optimize treatment pathways and ultimately support better patient outcomes, future real-world evidence studies and patient-focused drug development should consider the aspects of eBC treatment and disease management that matter most to patients.

## Figures and Tables

**Table 1:** Sociodemographic and Clinical Characteristics of Study Participants.

Characteristic	Total (*n* = 36)	Disease Stage		
		I (*n* = 18)	II (*n* = 11)	III (*n* = 7)
**Age**	*n* = 36	*n* = 18	*n* = 11	*n* = 7
Mean (SD)	49.9 (9.49)	50.6 (11.03)	51.0 (7.72)	46.3 (7.93)
Median	50.5	51.0	52.0	45.0
IQR	42.8–56.0	43.0–56.8	44.0–56.5	42.5–52.0
Min, max	28, 66	28, 66	41, 62	34, 56
***BRCA* mutations, *n* (%)**	*n* = 36	*n* = 18	*n* = 11	*n* = 7
Yes	5 (14)	0	3 (27)	2 (29)
No	21 (58)	13 (72)	5 (45)	3 (43)
Do not know	10 (28)	5 (28)	3 (27)	2 (29)
**Racial background, *n* (%)**	*n* = 35	*n* = 18	*n* = 10	*n* = 7
White	22 (63)	12 (67)	5 (50)	5 (71)
Black or African American	8 (23)	3 (17)	3 (30)	2 (29)
Asian	1 (3)	0	1 (10)	0
Native Hawaiian or other	2 (6)	2 (11)	0	0
Prefer not to respond	2 (6)	1 (6)	1 (10)	0
**Ethnicity, *n* (%)**	*n* = 35	*n* = 18	*n* = 10	*n* = 7
Hispanic or Latino	6 (17)	1 (6)	1 (10)	4 (57)
Not Hispanic or Latino	28 (80.0)	16 (89)	9 (90)	3 (43)
Prefer not to respond	1 (3)	1 (6)	0	0
**Education, *n* (%)**	***n* = 35**	***n* = 18**	***n* = 10**	***n* = 7**
Primary school	0	0	0	0
Secondary school	7 (20)	1 (6)	5 (50)	1 (14)
Associate degree	1 (3)	1 (6)	0	0
Some college	12 (34)	9 (50)	0	3 (43)
College/university	9 (26)	5 (28)	2 (20)	2 (29)
Postgraduate degree	6 (17)	2 (11)	3 (30)	1 (14)
Other	0	0	0	0
**Frequency of needing help with medical information, *n* (%) ^a^**	*n* = 35	*n* = 18	*n* = 10	*n* = 7
Never	18 (51)	8 (44)	7 (70)	3 (43)
Rarely	11 (31)	8 (44)	1 (10)	2 (29)
Sometimes	6 (17)	2 (11)	2 (20)	2 (29)
Often	0	0	0	0
Always	0	0	0	0
**Breast cancer subtype, *n* (%)**	*n* = 36	*n* = 18	*n* = 11	*n* = 7
HR+/HER2-	10 (28)	5 (28)	5 (45)	0
HER2+	14 (39) ^b^	6 (33)	4 (36)	4 (57)
TNBC	12 (33)	7 (39)	2 (18)	3 (43)

Abbreviations: HER2- human epidermal growth factor receptor 2-negative; HER2+ human epidermal growth factor receptor 2-positive; HR+ hormone receptor-positive; IQR interquartile range; SD standard deviation; TNBC triple-negative breast cancer. ^a^ Evaluated using the following question: “How often do you need to have someone help you when you read instructions, pamphlets, or other written material from your doctor or pharmacy?” ^b^ Three of these participants had HR+/HER2+ eBC.

## Data Availability

Data is contained within the article or Supplementary Material.

## Supplementary Materials

The following supporting information can be downloaded at https://www.mdpi.com/article/10.3390/cancers17213514/s1. Table S1. Illustrative Patient Quotes: Diagnosis and Genetic Testing, Signs/Symptoms, and Treatment Experiences. Table S2. Illustrative Patient Quotes: Impacts. Table S3. Illustrative Patient Quotes: Unmet Needs. Table S4. COREQ checklist. Table S5. Saturation Grids for Signs and Symptoms and Impacts. Figure S1. Signs and Symptoms Experienced by Patients with Early-Stage Breast Cancer. Figure S2. Endorsement of Signs/Symptoms by Disease Stage and Breast Cancer Subtype. Figure S3. Early-Stage Breast Cancer Treatments Received Before and at the Time of the Interviews. Figure S4. Side Effects of eBC Treatment as Reported by Participants. Figure S5. Endorsement of Treatment Side Effects by Disease Stage and Breast Cancer Subtype. Figure S6. Impacts Experienced by Patients with Early-Stage Breast Cancer. Figure S7. Endorsement of Impacts by Disease Stage and Breast Cancer Subtype.
