# Supplementary material for: Exploring the Priorities of Patients with Early Breast Cancer in the United States: A Qualitative Interview Study and Patient-Informed Conceptual Disease Model
Source: Cancers (Basel). 2025 Oct 31;17(21):3514. doi: 10.3390/cancers17213514 (PMC12607628; doi:10.3390/cancers17213514)
Supplement: Supplementary file 1 [file cancers-17-03514-s001.zip › cancers-3893365-supplementary.pdf]

Exploring the Priorities of Patients with Early Breast Cancer in the United States: A Qualitative Interview Study and Patient-Informed Conceptual Disease Model

Ashley Duenas; Zulikhat Segunmaru; Deborah Collyar; Debora Denardi; Claudine Clucas; Klaudia Kornalska; Qixin Li; Chintal H. Shah; Paul Swinburn; Mariana Chavez-MacGregor; Xiaoqing Xu

Corresponding author: Ashley Duenas

PPD Evidera Patient Centered Research, Thermo Fisher Scientific, London, UK

ashley.duenas@thermofisher.com

SUPPLEMENTARY MATERIALS

Supplementary Methods

Online Sociodemographic and Medical Background Questionnaire

This questionnaire included the following sections: Medical Background, Sociodemographic Questions, and EQ-5D-5L. Data collected as part of each section is presented below.

Medical Background

- Stage of breast cancer
- Status of first vs re-occurring breast cancer diagnosis
- Date of the first and most recent (if applicable) breast cancer diagnosis
- Tumor type of breast cancer
- BRCA mutation status
- History of gynecological cancers
- Family history of breast cancer
- Weight
- Treatments completed before the interview
- Types of surgery completed before the interview
- Treatments received at the time of the interview
- Types of surgery planned at the time of the interview
- Co-morbidities
- Most bothersome symptoms
- Most bothersome impacts

Sociodemographic Questions

- Age

- Gender
- Ethnic background
- Racial background
- Urban vs rural status
- Highest education status
- Employment status
- Health insurance status
- Marital status
- Received support from individuals
- Number of individuals living in the participant's household
- Combined household income
- Health literacy (help with reading medical documents)

EQ-5D-5L

- EQ-5D-5L domains
- VAS score

Semi-Structured Interview Guide

Below are topics covered in the interview guide, with example questions. Following the first three interviews, modifications were made to the interview guide. The wording of the questions was amended to increase clarity, and the number of questions was reduced to allow more in-depth answers. While the final interview guide included up to 52 unique questions, the flow of the questions was adapted for each interview based on participant responses.

EBC Diagnosis and Treatment

- Can you please talk me through your journey with eBC from diagnosis until now?

  [Interviewer]: *If not mentioned spontaneously, please probe:*

  - I see you were diagnosed with breast cancer in [report timeframe from screening form], can you briefly walk me through your diagnoses? What was that experience like for you?
  - Briefly describe any treatments or procedures you had for eBC?

  [Interviewer] *to probe on list treatments and surgeries the participant reported in the survey if not spontaneously mentioned]. Are you currently receiving treatment now?*

  - Did you feel you were given enough information about different treatment options?
  - How is your current treatment regimen working for you?
- Did you have a genetic test after your diagnosis? Did you understand why this was being asked of you? Please explain.
  - Did you receive any genetic counseling with the test?
  - Can you explain how your genetic test results helped inform your treatment decisions?
  - Were there any challenges that you experienced as a result of taking the test? Please provide an example.
  - ***[Interviewer]:*** *If the patient did not have a genetic test, ask if they think it would be important to have this? How would that type of information be useful? NOTE: keep in mind some patient may not want to know or understand the results.*

EBC Symptoms

- *Have you ever experienced any symptoms in relation to eBC, or the received treatments?*
- So far, you listed ***[Interviewer]****: list symptoms that the participant reported*. Which of these symptoms would you say is the most important to you? Why is it the most important one?
- Are you still currently experiencing that symptom?
- Have you experienced any other symptoms in relation to your diagnosis of eBC? Were any of these symptoms related to the treatment?

[Interviewer: probe for each of the symptoms mentioned as most important]

[Interviewer]: *If the following were not mentioned spontaneously, probe for the following specific symptoms:*

- Do you currently experience any breast symptoms? Can you please tell me more about it? *If not:* Did you experience breast symptoms in the past?
- Do you currently experience any pain/discomfort? Can you please tell me more about it? *If not:* Did you experience pain/discomfort in the past? Do you feel this is due to the disease specifically? Or treatment? Or other cause?
- Do you currently experience any problems or concerns in terms of sexual function? Please describe. *If not:* Did you experience any problems with sexual function in the past?
- Do you currently experience any problems with being able to concentrate or remember things? Please describe. *If not:* Did you experience problems with cognitive function in the past?

- Do you currently experience fatigue? How often do you feel fatigued? How often do you feel a lack of strength or low energy? Do you consider fatigue to be different from low energy? From lack of strength? How so? *If not:* Did you experience fatigue in the past?
- What would you like to be different in terms of the symptoms that you experience with eBC or the treatments for eBC?

EBC Impacts

Now I would like to hear more about how the diagnosis of EBC, and the previous and current treatments you received played a role in your daily life.

[Interviewer]: *If impacts came up in previous section, summarize or mirror back to the patient what was stated and probe further.*

- Could you tell me what type of impacts you experience as a result of EBC diagnosis and treatments?
    - By impact I mean, how do the experiences and symptoms of EBC diagnosis and treatment affect your everyday life?
    - ***[Interviewer]****: listen for the specific wording the participant uses to describe their experiences, for example, fatigue impacting on daily activities or work*]

[Interviewer]: *If not mentioned spontaneously, probe into the following impacts; however, focus primarily on impacts* ***in bold****. Due to the time available in the interview, not all impacts may be probed. Also, refer back to answers given on the pre-interview survey and further probe on the top three impacts they listed.*

How does diagnosis and treatment of EBC impact:

- How you feel physically, e.g., being able to move/walk/lift objects?
- Weight changes (gain or loss)?

- Daily activities such as washing and dressing, getting groceries or getting to work?
- Lifestyle (e.g., exercise and nutrition)?
- Your ability to fulfill roles and responsibilities important in your life, such as caring for your children or interacting with your family/friends?
- How you feel emotionally (e.g., feeling sad, feeling nervous, and/or feeling withdrawn)?
- Psychological aspects (e.g., finding purpose, motivation, and outlook on life)?
- Body image and body satisfaction?
- Ability to concentrate and remember things?
- Social life and relationships (e.g., friendships, relationships with family or significant other, marital relationships)?
- Sexual functioning (e.g., enjoyment, distress) and intimacy?
- Work/School (e.g., difficulty working, taking time off for treatment pressure to return to work or return to work early after treatment, working during treatment, thoughts on early retirement, cancer discrimination at work)?
- Financial aspects (e.g., financial stress or difficulties)?
- Anything else?

Overall Experience and Unmet Needs

[Interviewer]: *Mirror back what participant discusses up to this point and begin to probe on which aspects are most important.*

- What aspect of your experience with EBC diagnosis and treatment has been most significant or impactful? Why is that?
- How would you describe an improvement in your condition that would make a real difference in your everyday life?

- Do you feel that there are aspects of your treatment experience that could be supported better?
- Which aspects? In what ways? How so?

[Interviewer]: Probe the participant on the following:

- *Information about treatment and complications*
- *Emotional needs*
- *Financial support and funding for treatment, treatment access*

End of Interview

Thank you so much for your time today. We really appreciate your help. Before we close, is there anything else you would like to add that we may have missed in today's conversation?

Thank you for your time today. Your input has been very helpful. We have completed the interview.

Coding Framework

The coding framework covered key themes related to eBC diagnosis and treatment, symptom experiences, side effects, impacts, and unmet needs. For all symptoms, side effects, and impacts that were reported, additional sub-codes were added to include a description of what was reported and whether it was spontaneous or probed. In total, there were approximately 485 codes in the final codebook.

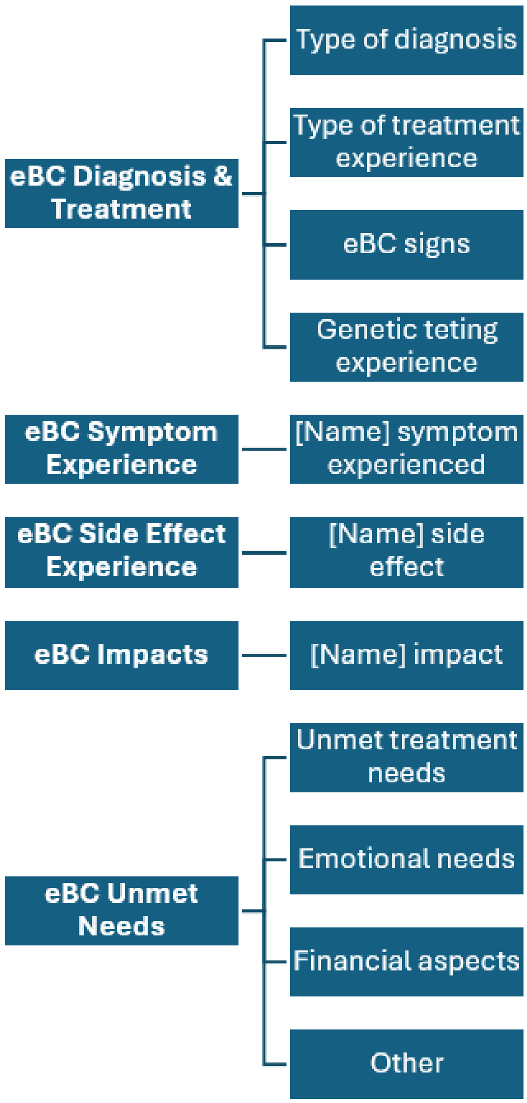

Supplementary Tables

Table S1. Illustrative Patient Quotes: Diagnosis and Genetic Testing, Signs/Symptoms, and Treatment Experiences

**Diagnosis and Genetic Testing**<br>• "*Oh gosh, the diagnosis hit me like a ton of bricks.*" – P16_Stage I_HER2+<br>• "*Well, I went to the doctor for a regular mammogram and when I went for the mammogram, that's when it was discovered. I was kinda shocked, and so there was a lot of discussion around that, with the different treatments that I could have. So, my doctor wanted me to do chemo first. […] So, everything went pretty quickly, so it's still overwhelming, the whole process of everything that is happening. It's just all still unbelievable*" – P34_Stage I_TNBC
**Signs/Symptoms**<br>• "*Well, I would say that it was like a marble, kind of I guess, the size of a marble.*" – P4_Stage III_HER2+<br>• "*Okay. So, the early onset breast cancer, I had a strange itching in my breast and that was the pressure of the lump. I was exhausted all the time. I didn't know why. I had pain; it felt like my underarm was on fire all the time, like shooting pain.*" – P12_Stage III_HER2+
**Treatment Experiences**<br>• "*Okay. The double mastectomy, it was a lot. My downtime was long because they did the DIEP flap, which was removed from my stomach and created my new breast. It was just a different experience. You know, just the whole process was a lot just to take and very painful […] the pain that I went through was severe for me.*" – P8_Stage I_HR+/HER2-<br>• "*…I was given the option to have radiation in several different increments. I could go for 6 or 8 weeks, 3 days a week. If you can compact it, you'll be much happier. I mean doing 5 days was a lot but being done in one week just made me feel great, I was done.*" – P1_Stage I_HR+/HER2-<br>• "*I had several weeks of radiation. It was on my right breast. It was horrible. Not horrible, but those weeks and months were just so tiresome for me. The radiation drained me a lot, and I didn't have no emotional people. I didn't even really let nobody know, so I kind of battled it all by myself because my mom, she's older and she has health problems. Sometimes, I would have them drive me if I was too weak to go on my own, but they didn't know the extent of all of it.*" – P36_Stage I_TNBC

Table S2. Illustrative Patient Quotes: Impacts

Emotional and Psychological Impacts	• "*Unfortunately, impacted a lot that my life and I'm not that happy person anymore because I'm afraid that somehow this chemo does not work, or even if it worked, it might come back because I've seen some patients, I've heard, for example, from friends, family, maybe that some patients have got it back and it's harder for them […] So, it brought me a lot of anxiety and depressed and chaos and these things.*" – P13_Stage I_HER2+<br>• "*I think it's just been more emotional on me, it's heavy, it's where your mind... I now have a... I get sidetracked, because it's what... I take a medication every day, I'm going to more doctor's appointments, I'm being observed, so it's a hot topic of my life […] So, it's very eye-opening for not just myself, my family, my friends, it's very... very debilitating.*" – P3_Stage II_HR+/HER2-
Social Life	• "*So, socially, I almost feel like I haven't… I mean, I've got a couple friends. I might get together with one of them every once in a while and we'll go get lunch or something. But, aside from that, I don't really feel like I've been very social. So, yeah, I think it has impacted me socially. I don't interact as much as I used to.*" – P10_Stage I_HR+/HER2-<br>• "*I like to just curl up on the bed or on the couch and read or go to audio books, watch movies 'cause what do you talk about? I mean, when I go to friends' houses or family houses what's the first thing they start talking about? It's my health issues or their health issues.*" – P11_Stage I_HER2+
Body Satisfaction	• "*…once I reached a certain age my entire progress of my physical maturation… the two things I always wanted was my long blonde hair because I loved that and when I finally got breasts that I was comfortable with. To me it's almost ironic and comical that if you had said to me "Are there any things about your body you wanted to keep?", those would be the two. I would have been like, "My breasts and my hair". So, that's kinda more my body perception.*" – P28_Stage I_TNBC<br>• "*I had my energy back, but then I had this huge burn across my chest and the wedding dress showed my chest, so I was really self-conscious about the burn, and I had no hair, so that was really embarrassing to me.*" – P12_Stage III_HER2+
Daily Activities	• "*Doing my chores, as far as my household chores, I don't do them as well and I don't put as much effort into doing them as I used to. It's because I just don't feel physically up to it.*" – P16_Stage I_HER2+<br>• "*Washing and getting myself dressed and stuff, that does take me more time than it did. It's not I can just do without any problem. I do have a little bit of problems getting myself dressed and then the activities… I don't… Around the house and stuff like that, I don't… I don't do as much as I used to and all in one day. I would say, for example, on my*

	cleaning day where I would go through and just start at one end of the house and do the whole house. Now I don't, I divide it up three or four days." – P25_Stage III_TNBC
Physical Functioning	• "*Well, with my exercising there is… I don't want to do as much exercise as I normally used to do. So, I have to be able to get my mind ready for the day to do more exercise like I used to do. I used to do power walking; I can't really do that in the mornings now. Also, maybe sometimes when I'm going to the grocery store I have to plan the day, instead of just going randomly. I can't do that at all.*" – P5_Stage I_HR+/HER2-
Sexual Functioning	• "*My libido has really went down. I am not as active as I was. It's just like I don't have the desire to do… you know, to have sex…*" – P23_Stage II_HER2+<br>• "*…so me and my husband to be honest did not have sex for about a year. … it's at the point and it's nothing to do with him at all. It is entirely that I have zero sexual desire or interest, but it has also progressed into a discomfort with even me in my own body right now.*" – P28_Stage I_TNBC
Lifestyle	• "*Well, everything in my life has kinda come to a stop right now. I usually exercise like crazy. That's not happening.*" – P15_Stage I_HER2+<br>• "*I think because since early November when all of this occurred, I have not necessarily been myself where I am as outgoing and active […] I've been very much… I don't want to say like a couch potato, but I've been pretty boring.*" – P21_Stage II_HER2+
Cognitive Functioning	• "*The first year, I couldn't really remember a lot. […] I'm still forgetful at times, but not as much as I was that first year.*" – P8_Stage I_HR+/HER2-<br>• "*I'm not as focused as before. I think there's a lot on my mind, maybe. Sometimes I feel like, yeah, I'm lost. I can't focus on something. I can't keep track of what I want to do.*" – P6_Stage I_HR+/HER2-
Intimacy	• "*At first, when I was diagnosed, it was fine but once I started going through the treatment, it started taking an effect on my energy and everything else. It kinda… the sex life just went down the drain and even after that, it's up and down and I haven't had the feeling to even wanna do that.*" – P23_Stage II_HER2+<br>• "*My husband is a million times supportive, always, but intimacy and things like that, it all starts changing because of how I feel about myself, how I feel about my body. Sitting in bed, laying there, thinking my body betrayed me. My tits are trying to kill me, feels pretty strange, and so yeah, I feel like intimacy was definitely an issue.*" – P7_Stage II_HER2+
Work and School	• "*So, from the day I did my biopsy, I couldn't go back to work the next day. I was working as a teacher assistant in a daycare. Since my arm was sore and I had some pain in the biopsy area, I asked them for a day off the next day and then the day after I got the result of the*

	biopsy. And from that day, I haven't gone back to work. I got so busy with appointments, mammograms, ultrasounds, and then oncologists. My life was about appointments every single day, so I was going to hospitals almost every day for sometimes even two or three appointments a day." – P6_Stage I_HR+/HER2-
<u>Role Functioning</u>	• "*…my mom actually has been a big help. I wouldn't know what to do without her. Then sometimes she can't help […] My girls will help with washing the dishes, and everyone pretty helps and whatnot. But especially the days of my treatment, I just don't have any energy at all.*" – P34_Stage I_TNBC<br>• "*I was so busy with just going to doctor's appointments. I even told my kids, "I'm so busy now, I can't…" I wasn't able to take them out. I wasn't able to attend their activities, study with them; I was just focused on health.*" – P6Stage I_HR+/HER2-

Table S3. Illustrative Patient Quotes: Unmet Needs

- "*I think a whole plan in reference to health insurance […] I think that needs to be laid out a lot because a lot of it is not laid out when you're going through it. It's just that your mind is focused on doing the treatment and trying to get well. You don't really focus on that part, you just know that you have insurance, but you just wanna make sure that everything is covered and that you're getting what you need to make sure that you're not stressing and worrying because you're trying to get through treatment.*" – P23_Stage II_HER2+
- "*I feel that with chemotherapy, and maybe even radiation, you should have an advocate because not everybody's family can be there. And it's already physically exhausting to be there. So, if you could have just a member of staff… they don't have to be a nurse. They don't have to be anybody medically educated. Just someone who can sympathize with you and just sit there for the hour that you have to suffer through chemotherapy.*" P12_Stage III_HER2+

Table S4. COREQ checklist

Item	Guide questions/description	Page(s)
**Domain 1: Research team and reflexivity**		
Personal Characteristics		
1. Interviewer/facilitator	Which author/s conducted the interview or focus group?	4
2. Credentials	What were the researcher's credentials? E.g. PhD, MD	4
3. Occupation	What was their occupation at the time of the study?	4
4. Gender	Was the researcher male or female?	4
5. Experience and training	What experience or training did the researcher have?	4
Relationship with participants		
6. Relationship established	Was a relationship established prior to study commencement?	4
7. Participant knowledge of the interviewer	What did the participants know about the researcher? e.g. personal goals, reasons for doing the research	4
8. Interviewer characteristics	What characteristics were reported about the interviewer/facilitator? e.g. Bias, assumptions, reasons and interests in the research topic	4
**Domain 2: Study design**		
Theoretical framework		
9. Methodological orientation and Theory	What methodological orientation was stated to underpin the study? e.g. grounded theory, discourse analysis, ethnography, phenomenology, content analysis	5
Participant selection		
10. Sampling	How were participants selected? e.g. purposive, convenience, consecutive, snowball	3
11. Method of approach	How were participants approached? e.g. face-to-face, telephone, mail, email	3
12. Sample size	How many participants were in the study?	5
13. Non-participation	How many people refused to participate or dropped out? Reasons?	5
Setting		
14. Setting of data collection	Where was the data collected? e.g. home, clinic, workplace	3
15. Presence of non-participants	Was anyone else present besides the participants and researchers?	4
16. Description of sample	What are the important characteristics of the sample? e.g. demographic data, date	5-6

Data collection		
17. Interview guide	Were questions, prompts, guides provided by the authors? Was it pilot tested?	4
18. Repeat interviews	Were repeat interviews carried out? If yes, how many?	4
19. Audio/visual recording	Did the research use audio or visual recording to collect the data?	4
20. Field notes	Were field notes made during and/or after the interview or focus group?	4
21. Duration	What was the duration of the interviews or focus group?	4
22. Data saturation	Was data saturation discussed?	5, 7, 9, 13
23. Transcripts returned	Were transcripts returned to participants for comment and/or correction?	4
**Domain 3: Analysis and findings**		
Data analysis		
24. Number of data coders	How many data coders coded the data?	5
25. Description of the coding tree	Did authors provide a description of the coding tree?	5
26. Derivation of themes	Were themes identified in advance or derived from the data?	5
27. Software	What software, if applicable, was used to manage the data?	5
28. Participant checking	Did participants provide feedback on the findings?	5
Reporting		
29. Quotations presented	Were participant quotations presented to illustrate the themes/findings? Was each quotation identified? e.g. participant number	7-11
30. Data and findings consistent	Was there consistency between the data presented and the findings?	7-14
31. Clarity of major themes	Were major themes clearly presented in the findings?	7-14
32. Clarity of minor themes	Is there a description of diverse cases or discussion of minor themes?	7-14

Tong A, Sainsbury P, Craig J. Consolidated criteria for reporting qualitative research (COREQ): a 32-item checklist for interviews and focus groups. Int J Qual Health Care. 2007;19(6):349-57.

Table S5. Saturation Grids for Signs and Symptoms and Impacts

	Overall Sample (N=36)						Participants endorsing the concept		
	Group 1 (n=6)	Group 2 (n=6)	Group 3 (n=6)	Group 4 (n=6)	Group 5 (n=6)	Group 6 (n=6)	S	P	Total N (%)
**Signs and symptoms[a]**									
Lump on breast or underarm							16	2	18 (50)
Breast or nipple pain							8	2	10 (28)
Energy-related (fatigue, lack of strength, energy)							8	1	9 (25)
Nipple or breast inflammation/swelling							3	0	3 (8)
Nipple discharge							3	0	3 (8)
Nipple retraction							2	0	2 (5)
Body chills							1	0	1 (3)
Skin changes							3	0	3 (8)
Weight loss							1	1	2 (6)
Cognitive							1	0	1 (3)
Fever							1	0	1 (3)
Numbness							1	0	1 (3)
Itching							1	0	1 (3)
Dimple in breast							1	0	1 (3)
**New concepts, n (%)**	**8 (57)**	**3 (21)**	**1 (7)**	**1 (7)**	**0**	**1 (7)**	**-**	**-**	**14 (100)**
**Impacts[b]**									
Emotional/psychological							31	5	36 (100)
Social life							12	14	26 (72)
Body satisfaction							10	15	25 (14)
Daily activities							5	14	19 (53)
Physical functioning							10	7	17 (47)
Sexual functioning							2	11	13 (36)
Lifestyle							6	6	12 (33)
Cognitive functioning							1	10	11 (31)
Intimacy							3	8	11 (31)
Work or school							8	3	11 (31)
Financial							3	7	10 (28)
Role functioning							2	7	9 (25)
Weight							2	6	8 (22)
Sleep							5	0	5 (14)
**New concepts, n (%)**	**13 (93)**	**1 (7)**	**0**	**0**	**0**	**0**	**-**	**-**	**14 (100)**

S spontaneously, *P* probed

[a] Eight (57%) of the 14 sign/symptom concepts elicited were reported in the first six interviews. An additional three concepts emerged in the second set of six interviews and one concept each in the third, fourth, and sixth sets of six interviews. With the exception of a single report of a dimple in the breast in the final interview, all other symptoms were identified in the first four groups of interviews.

[b] Thirteen of 14 impact concepts (93%) were reported in the first six interviews. One new impact concept emerged in the second set of six interviews and none in subsequent interviews.

Supplementary Figures

Figure S1. Signs and Symptoms Experienced by Patients with Early-Stage Breast Cancer

Nine participants (25%) did not endorse any signs or symptoms

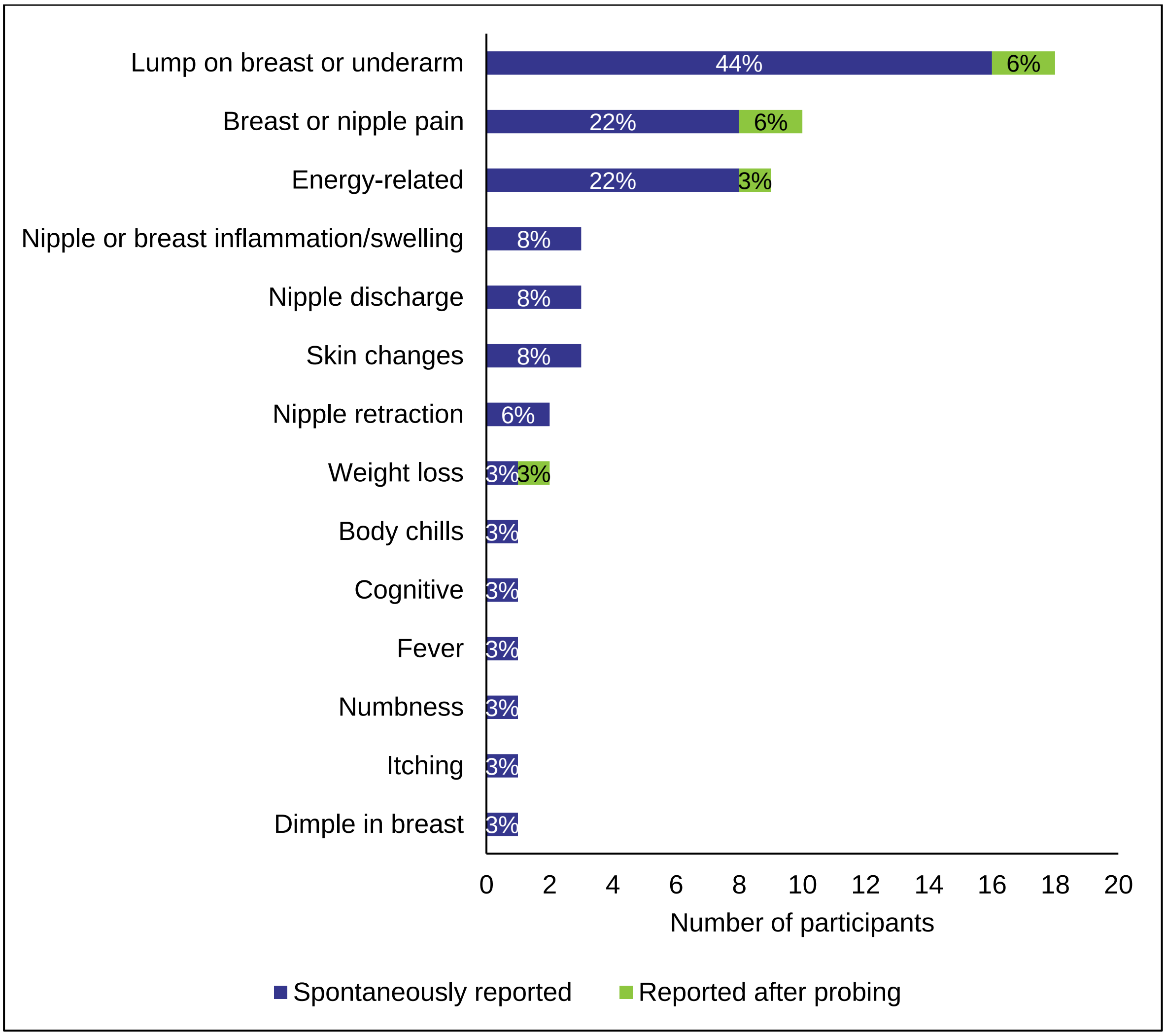

Figure S2. Endorsement of Signs/Symptoms by Disease Stage and Breast Cancer Subtype

A. Signs/symptoms by disease stage. B. Signs/symptoms by breast cancer subtype. Symptoms endorsed by only one participant (numbness, dimple in breast, cognitive symptoms) are not shown. *HER2-* human epidermal growth factor receptor 2-negative, *HER2+* human epidermal growth factor receptor 2-positive, *HR+* hormone receptor-positive, *TNBC* triple-negative breast cancer

A

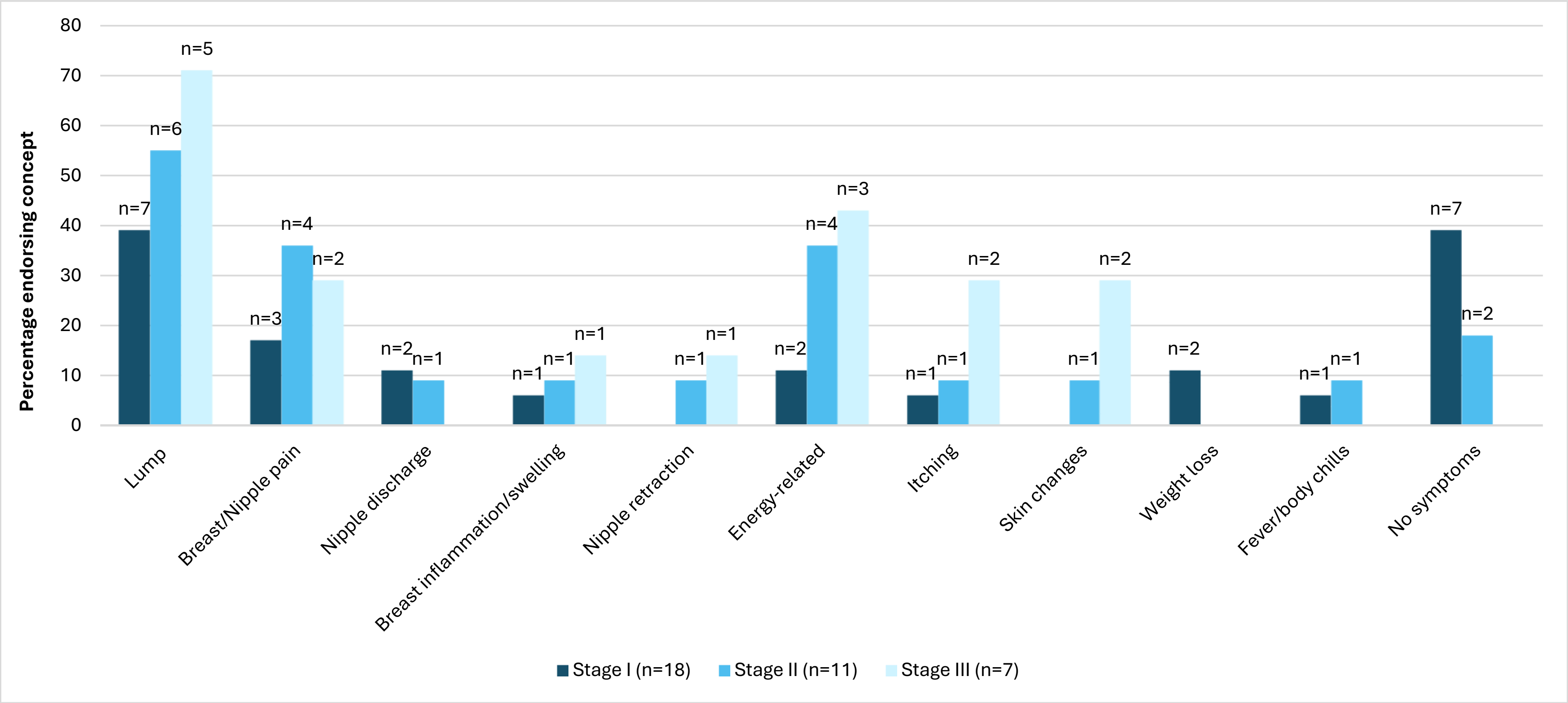

B

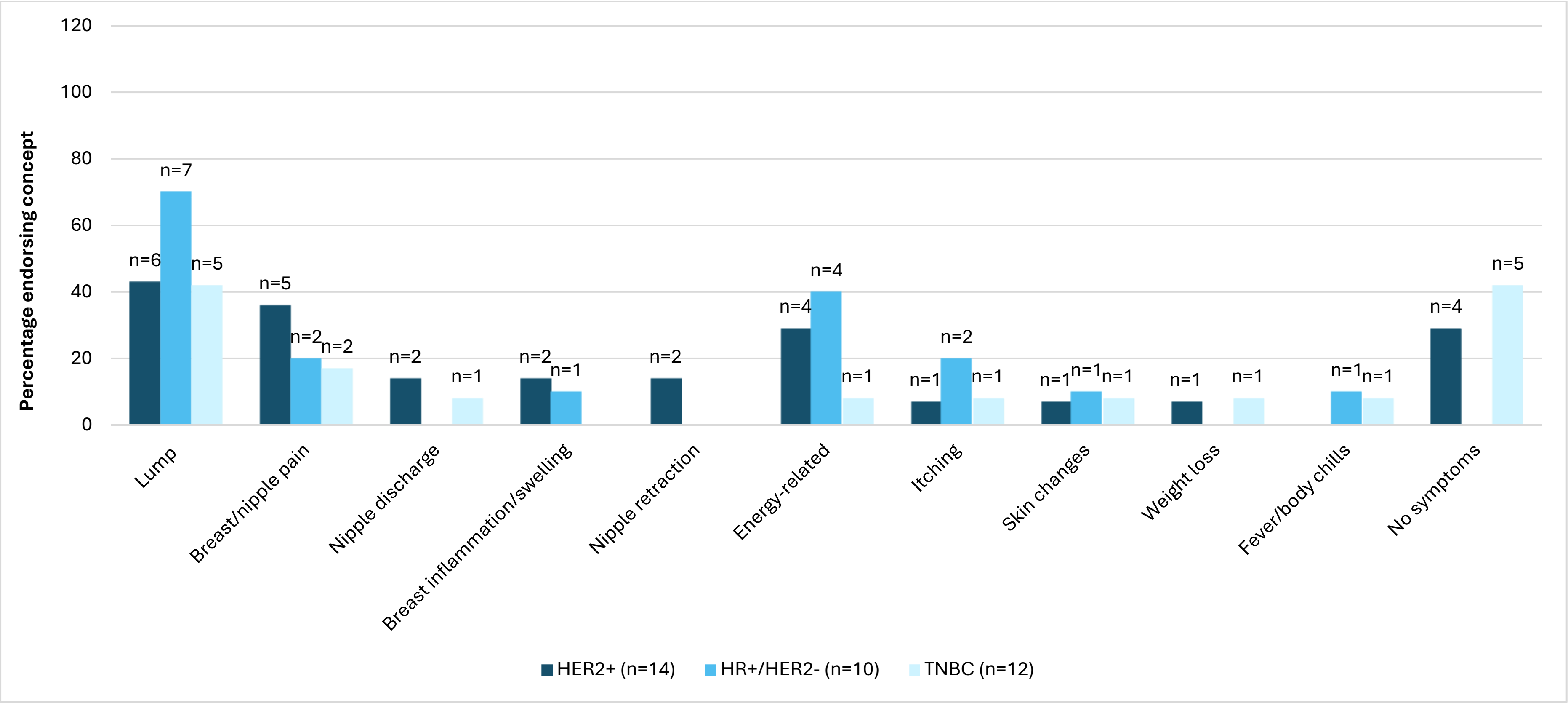
Percentage endorsing concept
120
100
80
60
40
20
0
n=6
n=7
n=5
n=5
n=2
n=2
n=2
n=1
n=2
n=1
n=2
n=4
n=4
n=1
n=1
n=2
n=1
n=1
n=1
n=1
n=1
n=1
n=1
n=1
n=4
n=5
Lump
Breast/nipple pain
Nipple discharge
Breast inflammation/swelling
Nipple retraction
Energy-related
Itching
Skin changes
Weight loss
Fever/body chills
No symptoms
HER2+ (n=14)
HR+/HER2- (n=10)
TNBC (n=12)

Figure S3. Early-Stage Breast Cancer Treatments Received Before and at the Time of the Interviews

*Participants waiting for a planned surgery at the time of the interview. HER2 human epidermal growth factor receptor 2

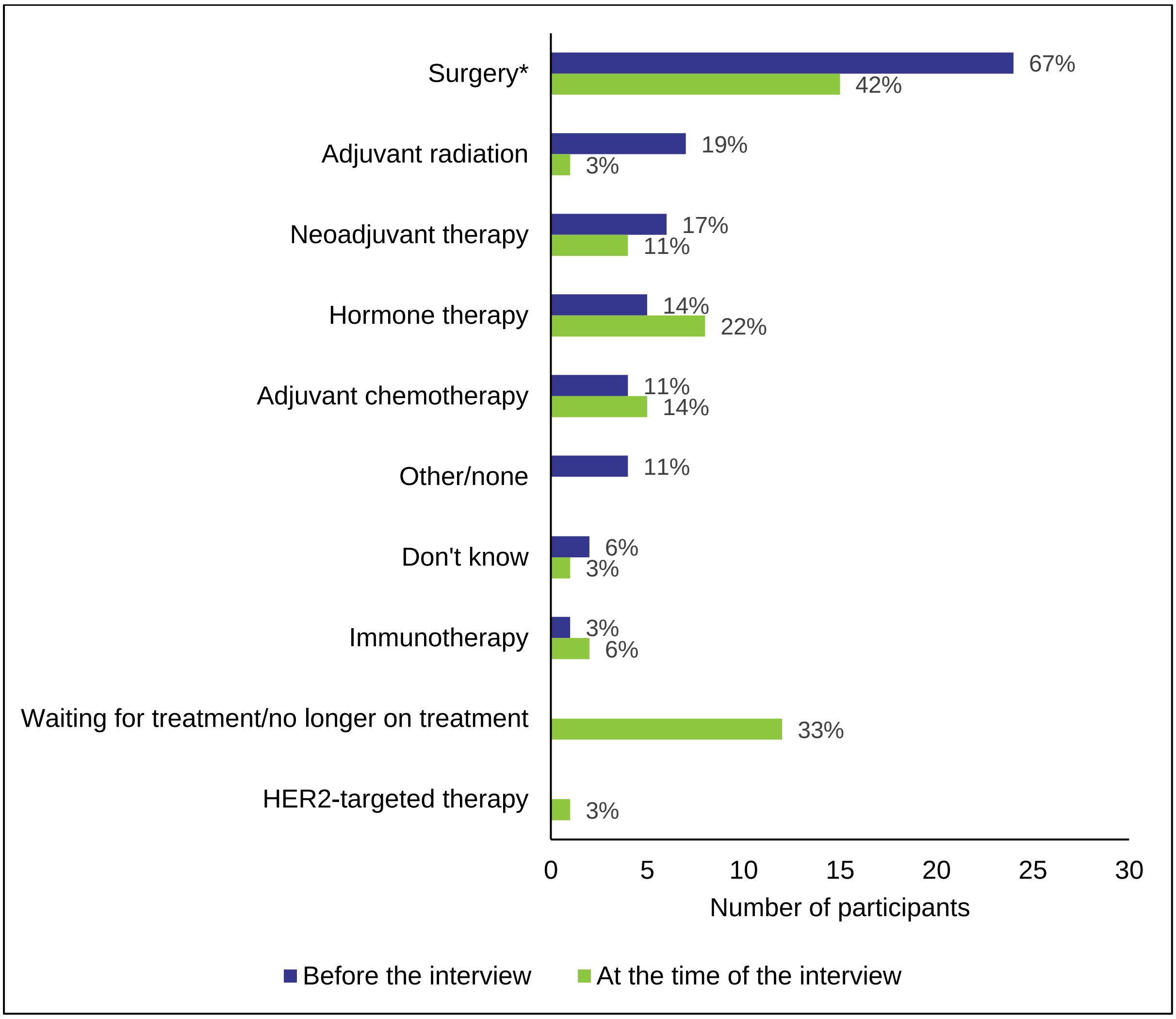

Figure S4. Side Effects of eBC Treatment as Reported by Participants

Side effects endorsed by ≤2 participants (anemia, arm symptoms, bone and skeletal, flu, lymphedema, neuropathy, shortness of breath/dyspnea, vision changes) are not shown. eBC early-stage breast cancer

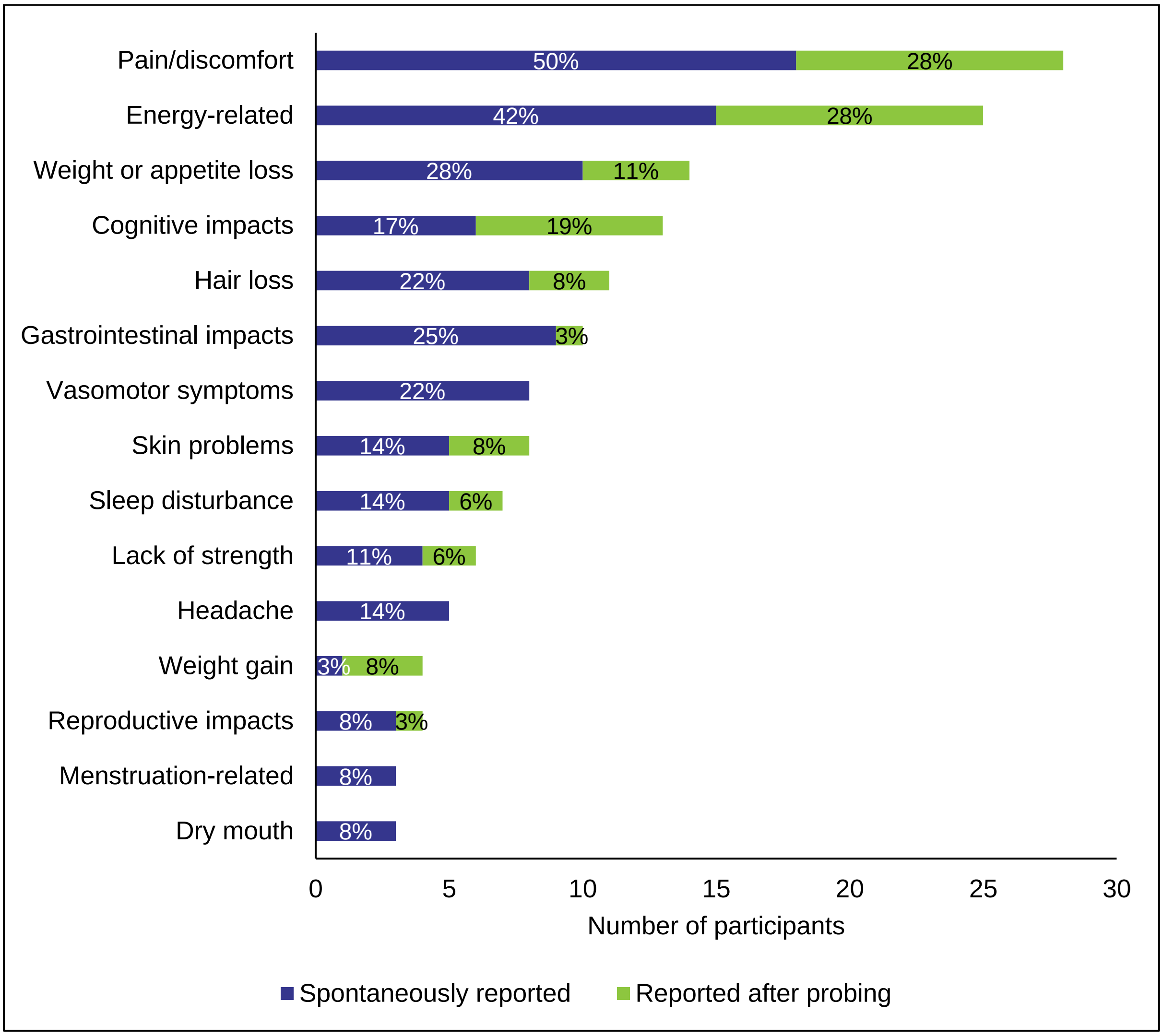

Figure S5. Endorsement of Treatment Side Effects by Disease Stage and Breast Cancer Subtype

A. Side effects by disease stage. B. Side effects by breast cancer subtype. *HER2-* human epidermal growth factor receptor 2-negative, *HER2+* human epidermal growth factor receptor 2-positive, *HR+* hormone receptor-positive, *TNBC* triple-negative breast cancer

A

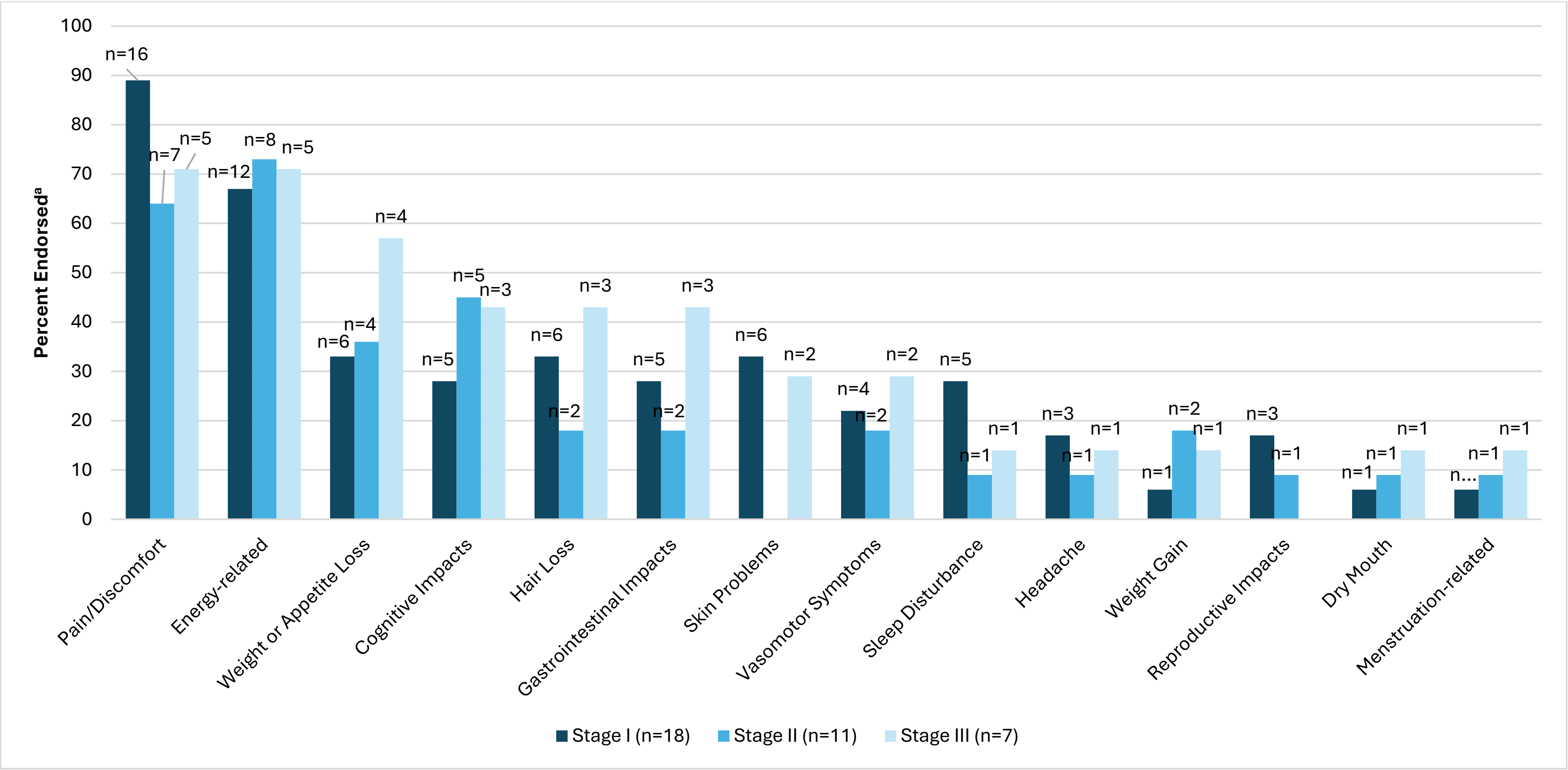

Percent Endorsed[a]
100
90
80
70
60
50
40
30
20
10
0
n=16
n=7
n=5
n=12
n=8
n=5
n=6
n=4
n=4
n=5
n=5
n=3
n=6
n=2
n=3
n=5
n=2
n=3
n=6
n=2
n=4
n=2
n=2
n=5
n=1
n=1
n=3
n=1
n=1
n=1
n=2
n=1
n=3
n=1
n=1
n=1
n=1
n…
n=1
n=1
Pain/Discomfort
Energy-related
Weight or Appetite Loss
Cognitive Impacts
Hair Loss
Gastrointestinal Impacts
Skin Problems
Vasomotor Symptoms
Sleep Disturbance
Headache
Weight Gain
Reproductive Impacts
Dry Mouth
Menstruation-related
Stage I (n=18)
Stage II (n=11)
Stage III (n=7)

B

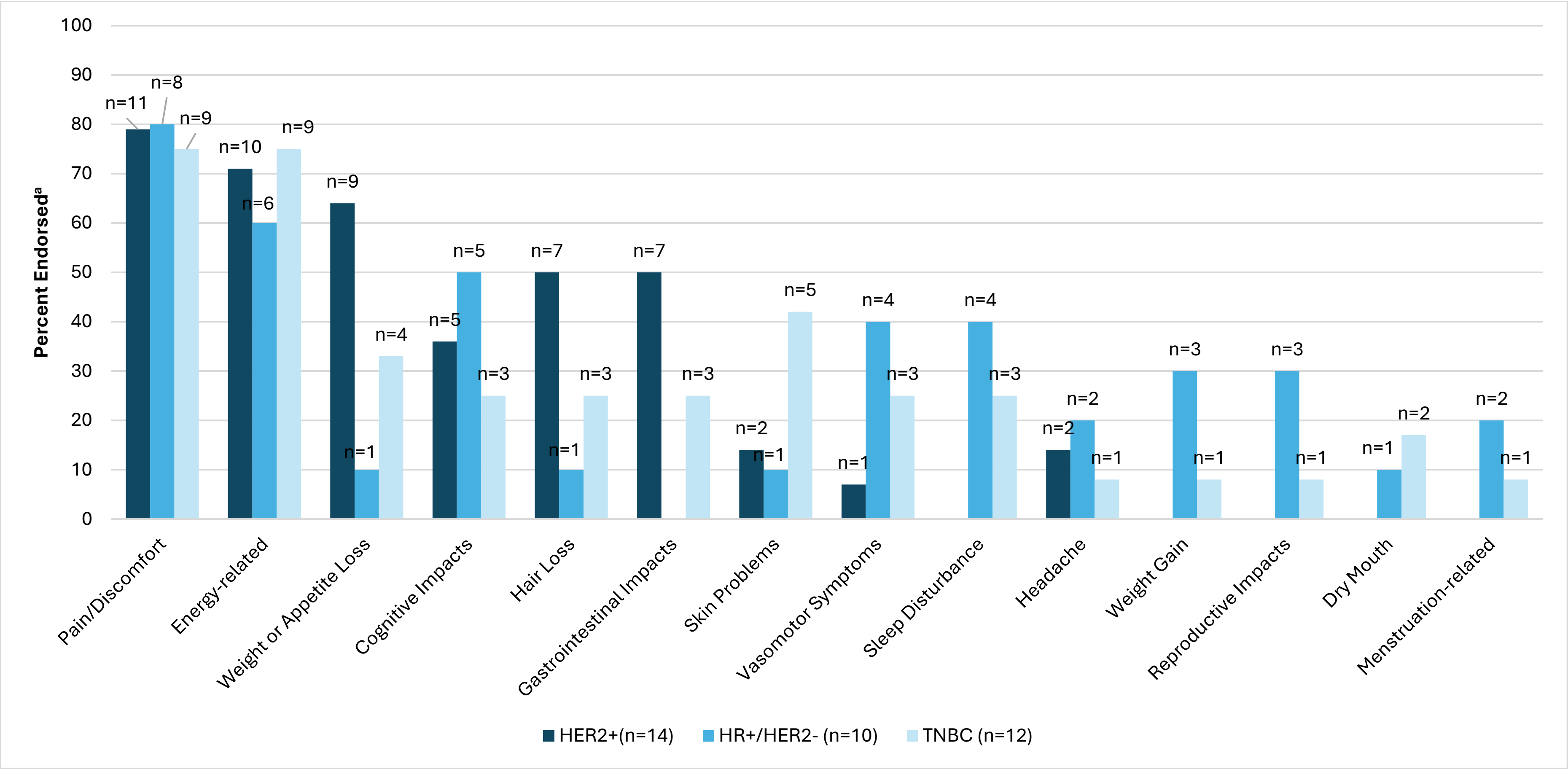

Percent Endorsed[a]
100
90
80
70
60
50
40
30
20
10
0
n=11
n=8
n=9
n=10
n=6
n=9
n=9
n=1
n=4
n=5
n=5
n=3
n=7
n=1
n=3
n=7
n=3
n=2
n=1
n=5
n=1
n=4
n=3
n=4
n=3
n=2
n=2
n=1
n=3
n=1
n=3
n=1
n=1
n=2
n=2
n=1
Pain/Discomfort
Energy-related
Weight or Appetite Loss
Cognitive Impacts
Hair Loss
Gastrointestinal Impacts
Skin Problems
Vasomotor Symptoms
Sleep Disturbance
Headache
Weight Gain
Reproductive Impacts
Dry Mouth
Menstruation-related
HER2+(n=14)
HR+/HER2- (n=10)
TNBC (n=12)

Figure S6. Impacts Experienced by Patients with Early-Stage Breast Cancer

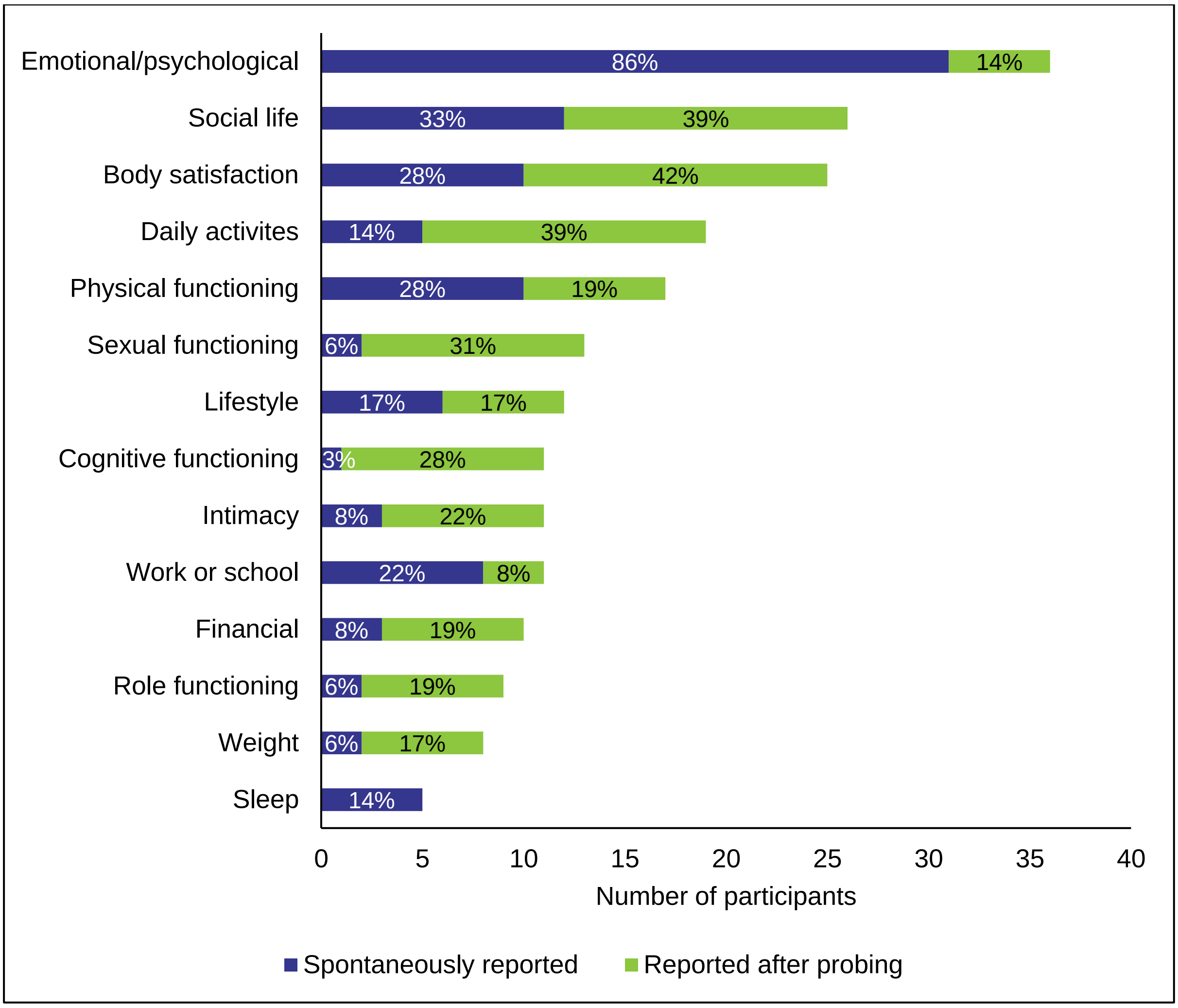

Figure S7. Endorsement of Impacts by Disease Stage and Breast Cancer Subtype

A. Impacts by disease stage. B. Impacts by breast cancer subtype. *HER2-* human epidermal growth factor receptor 2-negative, *HER2+* human epidermal growth factor receptor 2-positive, *HR+* hormone receptor-positive, *TNBC* triple-negative breast cancer

A

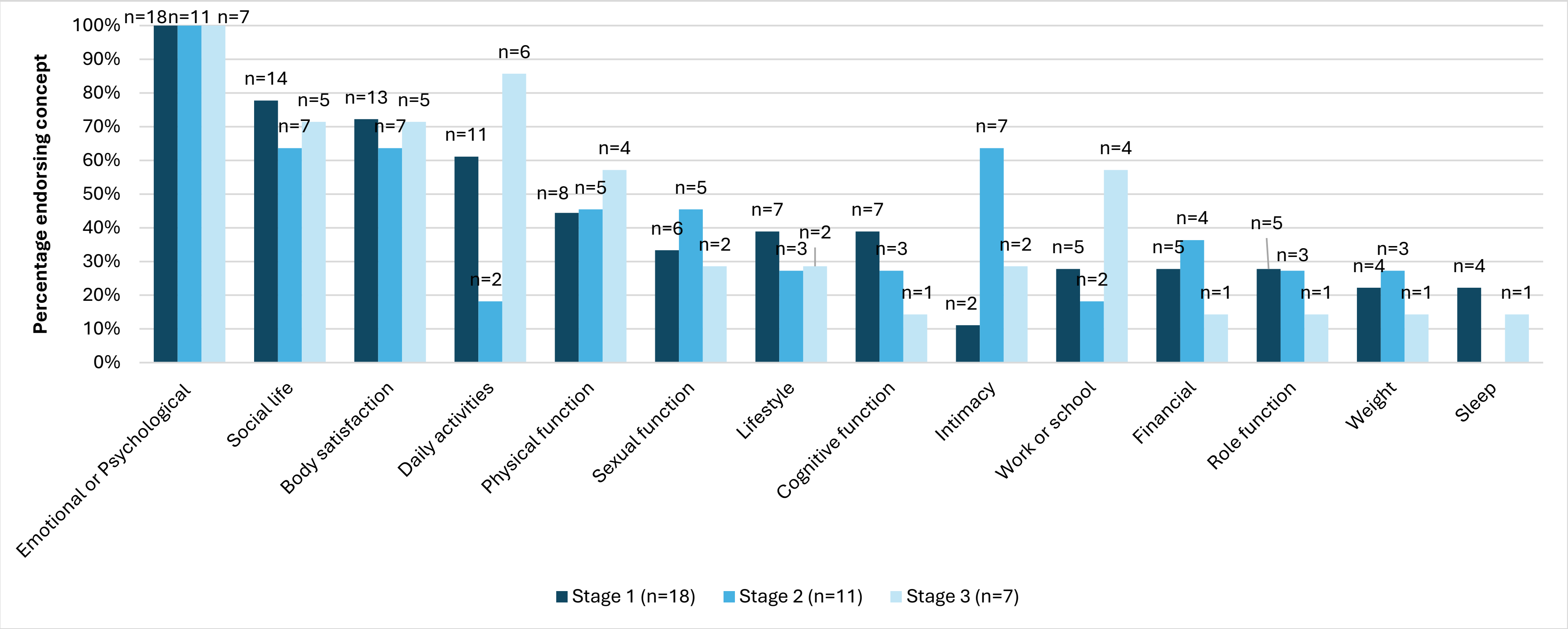

B

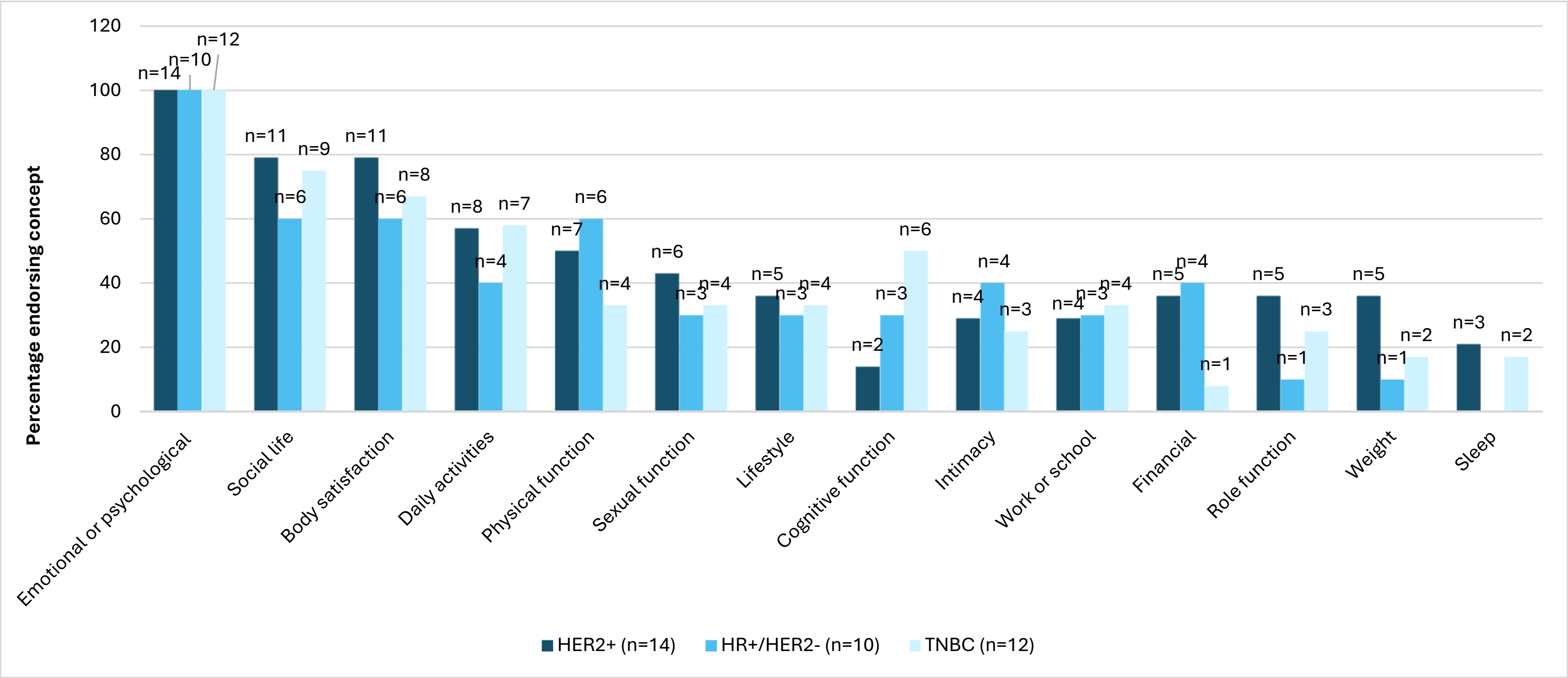

Percentage endorsing concept
120
100
80
60
40
20
0
n=14
n=10
n=12
n=11
n=6
n=9
n=11
n=6
n=8
n=8
n=4
n=7
n=7
n=6
n=4
n=6
n=3
n=4
n=5
n=3
n=4
n=2
n=3
n=6
n=4
n=4
n=3
n=4
n=3
n=4
n=5
n=4
n=1
n=5
n=1
n=3
n=5
n=1
n=2
n=3
n=2
Emotional or psychological
Social life
Body satisfaction
Daily activities
Physical function
Sexual function
Lifestyle
Cognitive function
Intimacy
Work or school
Financial
Role function
Weight
Sleep
HER2+ (n=14)
HR+/HER2- (n=10)
TNBC (n=12)
